# Genome-Wide Identification, Evolutionary Analysis, and Stress Responses of the *GRAS* Gene Family in Castor Beans

**DOI:** 10.3390/ijms17071004

**Published:** 2016-06-24

**Authors:** Wei Xu, Zexi Chen, Naeem Ahmed, Bing Han, Qinghua Cui, Aizhong Liu

**Affiliations:** 1 College of Life Sciences, Yunnan University, Kunming 650091, China; xuwei@mail.kib.ac.cn (W.X.); 15687385528@163.com (Z.C.); 2 Department of Economic Plants and Biotechnology, Yunnan Key Laboratory for Wild Plant Resources, Kunming Institute of Botany, Chinese Academy of Sciences, Kunming 650204, China; naeem.ahmed@uok.edu.pk (N.A.); hanbing@mail.kib.ac.cn (B.H.); 3 University of the Chinese Academy of Sciences, Beijing 100049, China; 4 Department of Botany, University of Karachi, Karachi-75270, Pakistan

**Keywords:** *GRAS* gene family, castor beans, gene expression, abiotic stress, phylogenetic relationship

## Abstract

Plant-specific GRAS transcription factors play important roles in regulating growth, development, and stress responses. Castor beans (*Ricinus communis*) are important non-edible oilseed plants, cultivated worldwide for its seed oils and its adaptability to growth conditions. In this study, we identified and characterized a total of 48 *GRAS* genes based on the castor bean genome. Combined with phylogenetic analysis, the castor bean GRAS members were divided into 13 distinct groups. Functional divergence analysis revealed the presence of mostly Type-I functional divergence. The gene structures and conserved motifs, both within and outside the GRAS domain, were characterized. Gene expression analysis, performed in various tissues and under a range of abiotic stress conditions, uncovered the potential functions of GRAS members in regulating plant growth development and stress responses. The results obtained from this study provide valuable information toward understanding the potential molecular mechanisms of GRAS proteins in castor beans. These findings also serve as a resource for identifying the genes that allow castor beans to grow in stressful conditions and to enable further breeding and genetic improvements in agriculture.

## 1. Introduction

The plant-specific *GRAS* gene family plays important roles in plant growth, development, and stress responses. The name GRAS is coined from the first three well-studied family members: GAI, RGA and SCR [1,2,3]. GRAS proteins typically range from 400–770 amino acid residues and contain a highly conserved sequence in their carboxyl termini. This conserved sequence is typically composed of several ordered motifs including LHR I, VHIID, LHR II, PFYRE, and SAW [4,5,6]. These conserved motifs significantly contribute to protein function [7,8], for instance, the mutation of PFYRE and SAW motifs in the RGA and SLR1 regions results in strong phenotypic changes in *Arabidopsis* [3,7]. The amino-termini of GRAS proteins are more divergent in length and sequence, suggesting their functional specificity [6].

With the rapid development of sequencing techniques, genome-wide scans for genes encoding GRAS domains have been conducted in many plant species, including *Arabidopsis* [6], rice [6], *Populus* [9], Chinese cabbage (*Brassica rapa* ssp. *pekinensis*) [10], *Prunus mume* [11], pine (*Pinus* *radiata*) [12], tomato (*Solanum lycopersicum*) [13], *Jatropha curcas* [14], and grapevine (*Vitis vinifera*) [15]. The *GRAS* gene family is generally divided into eight subfamilies—SCL3, SHR, PAT1, LISCL, DELLA, SCR, LS, and HAM—based on conserved motifs and sequence similarity. Each subfamily may have different functions in plant physiological processes [6]. For instance, the *Arabidopsis* *SCR* gene, the first identified GRAS member, is expressed in the cortex/endodermal initial cells and regulates the radial organization of the root [1]. Subsequent studies found that *Arabidopsis* SHR, another GRAS family member, is also essential for maintaining root development via a SCR/SHR complex [16,17]. There is evidence that AtSCL3 (SCARECROW-LIKE 3) can positively regulate the gibberellin (GA) pathway and functions as a repressor of DELLA, controlling GA homeostasis in root development [18,19]. Detailed studies on the regulatory mechanism of DELLA proteins have identified them as repressors of GA signaling [3]. The DELLA subfamily members including GAI, RGA, RGL1 (RGA-LIKE1), RGL2, and RGL3 are distinct from other GRAS proteins due to the conserved DELLA motif in their N-terminal region [20]. AtSCL13, belonging to the PAT1 subfamily, acts mainly as a positive regulator of the phytochrome B (phyB) signaling pathway [21], whereas other members within this subfamily such as PAT1 and SCL21 are involved in phytochrome A (phyA) signal transduction [21,22]. Rice MONOCULM 1 (OsMOC1), expressed in the axillary buds, plays an important role in the control of rice tillering [23]. Genes homologous to OsMOC1, in tomato (*Ls*) and *Arabidopsis* (*LAS/SCL18*), also function in the regulation of axillary meristem formation [24,25]. Some GRAS members such as NSP1 and NSP2 (or their protein complex) play a key role in nodulation, serving as possible regulators of Nod-factor-inducible gene expression in *Medicago truncatula* [26,27]. Furthermore, the NSP1 and NSP2 are required for strigolactone biosynthesis in *Medicago truncatula* and rice [28]. Recent studies have demonstrated that microRNA171 regulates several GRAS transcription factors including SCL6/SCL6-IV, SCL22/SCL6-III, and SCL27/SCL6-II in *Arabidopsis* [29,30,31]. In addition, GRAS members with stress-related functions have been found in *Arabidopsis* and cabbage (SCL13) [21,32], rice (CIGR1 and CIGR2) [33], tobacco (GRAS1) [34], and poplar (SCL7) [35].

The castor bean (*Ricinus communis* L. Euphorbiaceae) is an important non-edible oilseed crop. Its seed oils have a wide range of industrial applications [36], such as ideal feedstock for biodiesel production [37,38]. Increasing demand for castor bean oil, coupled with a strong ability to thrive in inferior growth conditions, has led to widespread castor bean cultivation in tropical, sub-tropical, and temperate regions. Breeding and improvement of castor beans have gained a great deal of attention in the scientific arena [39] and have prompted sequencing of the genome of the cultivated castor bean variety “Hale” [37]. The molecular mechanism underlying the adaptability of castor beans to grow under stressful conditions such as inadequate water and high temperatures remains unknown. Here, we investigated and characterized the *GRAS* gene family members based on the castor bean genome sequence. The expression profiles of these *GRAS* genes were examined among different tissues and a variety of stress conditions. These findings provide insight into the molecular functions of GRAS transcription factors in the castor bean plant.

## 2. Results

### 2.1. Identification of the GRAS Gene Family in Castor Beans

We identified a total of 48 distinct GRAS proteins based on a genome-wide search of the castor bean (Supplementary Table S1). To confirm these predicted proteins containing a bona fide GRAS domain, we evaluated them using SMART software [40] and analyzed their homologs in *Arabidopsis*. We found that all predicted proteins had a typically conserved GRAS domain, with the exception of one member (28166.m001040) that had two GRAS domains and two members (28966.m000535 and 29957.m001404) with partial domain sequences. The genes encoding proteins with partial domains were excluded from further analyses, while the other 46 members were considered as GRAS family members. These *GRAS* genes had open reading frames (ORFs) ranging from 1008 to 2532 bp and encoded proteins ranging from 335 to 843 amino acid (aa) residues in length. The molecular weights of those proteins ranged from 38 to 90 kDa, and most were weakly acidic, with a mean predicted theoretical pI (protein isoelectric point) of 5.8. In addition, we also searched for tandem duplication events in regions of GRAS members based on PTGBase analysis of the castor bean genome [41]. Tandem duplication events were identified in four different regions in nine GRAS members (28650.m000186 and 28650.m000187; 28650.m000189, 28650.m000190, and 28650.m000191; 29820.m000985 and 29820.m000986; 29889.m003284 and 29889.m003285).

To further validate the predicted *GRAS* genes in castor beans, we mapped and assembled genomic GRAS sequences to reads from our previous deep RNA-sequencing (RNA-seq) data [42]. A total of 28 genes were evaluated, with at least 10 reads coverage, and 25 showed nearly identical gene structures to predicted gene models. Three *GRAS* genes with discrepancies contained introns, despite reads mapped throughout the genes (29692.m000540 and 29916.m000544), or had an alternative splice site in the first intron and a misannotated second intron instead of the two predicted introns (29949.m000124) (Supplementary Figure S1). The corrected GRAS sequences were used for further analyses.

### 2.2. Phylogenetic Analysis and Functional Divergence of the GRAS Gene Families

**Figure 1:**
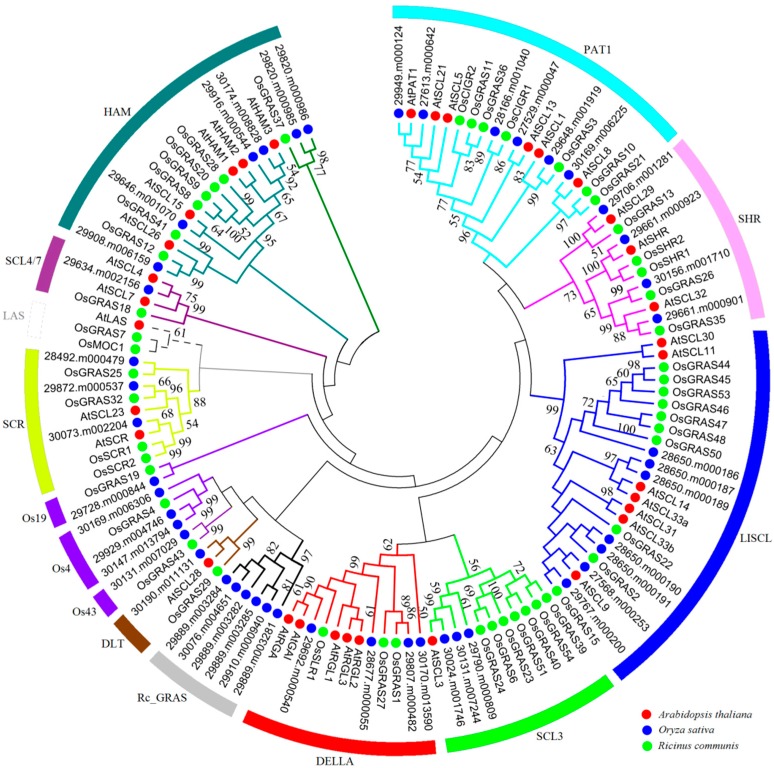
An unrooted phylogenetic tree of GRAS transcription factors from *Arabidopsis* *thaliana*, rice and castor beans. The multiple sequence alignment of GRAS proteins including *Arabidopsis* *thaliana* (red dots), castor beans (green dots) and rice (blue dots) were carried out using Clustal W [44], and the tree was generated with neighbor-joining methods. Members in the same clade were marked by the same color.

Generally, the conserved sequences within characteristic domain regions play an important role in the basic functions of transcription factors. Multiple sequence alignments of GRAS proteins in castor beans showed a low overall similarity in the N-termini regions but relatively conserved domain regions in the C-termini including the five characteristic GRAS-domain motifs: LHR I, VHIID, LHR II, PFYRE, and SAW (Supplementary Figure S2). To explore the evolutionary relationships within the *GRAS* gene family, 129 full-length proteins (46 from castor beans, 33 from *Arabidopsis*, and 50 from rice) were aligned to construct an unrooted tree with 14 distinct subfamilies (Figure 1). Consistent with previous studies, GRAS members from castor beans were found in 13 distinct clusters within the phylogenetic tree; specifically PAT1 (six members), SHR (four members), LISCL (seven members), SCL3 (three members), DELLA (four members), DLT (one member), Os43 (one member), Os4 (three members), Os19 (one member), SCR (three members), SCL4/7 (one member), HAM (six members), and Rc_GRAS (six members) subfamilies [6,12,13]. Interestingly, we found that the Rc_GRAS subfamily (including 29889.m003281, 29889.m003282, 29889.m003284, 29889.m003285, 29910.m000940, and 30076.m004651) contained only proteins from castor beans, whereas no LAS subfamily members were detected (Figure 1). Additionally, five GRAS members from castor beans lacked *Arabidopsis* homologs and were grouped with the rice-specific protein subfamilies “Os4”, “Os19”, and “Os43” previously reported [6]. The other nine subfamilies clustered with members of each species, indicating evolutionary and functional conservation among these subfamily members.

To test whether there is a functional divergence between GRAS subfamilies, we calculated the coefficients of Type-I (θ_I_) and Type-II (θ_II_) functional divergence using the DIVERGE (v3.0) software [43] that can rule out the influence of saturation of synonymous sites. The Type-I functional divergence is often used to examine the aa residues that occurred significantly at site-specific changes of evolutionary rates. Type-I functional divergence between two subfamilies means that some aa residues are highly conserved in one subfamily but highly variable in the other at same sites. The Type-II functional divergence is used to assess those residues that were due to site-specific shifts in aa properties. Type-II functional divergence between two subfamilies means that some aa residues are highly conserved in both subfamilies, but have different biochemical properties. The coefficients of functional divergence of Type-I (θ_I_) and Type-II (θ_II_) can reflect which factor is the main force for the functional divergence between any two GRAS subfamilies. In this analysis, six GRAS subfamilies were excluded because of their small size (Os4, Os19, Os43, LAS, DLT, and SCL4/7), and the remaining subfamilies were evaluated using the DIVERGE software based on the maximum mikelihood algorithm. Our results indicated that the coefficient of θ_I_, between any two subfamilies, was greater than 0 and varied from 0.33 to 0.98 (Table 1). Six pairs, LISCL/DELLA, LISCL/HAM, PAT1/HAM, SHR/LISCL, LISCL/SCR, and DELLA/HAM, were markedly divergent with statistically significant LRT (likelihood ratio test, *p* < 0.01). This is consistent with observations in the phylogenetic tree (see Figure 1), implying that some specific site changes may be subject to functional constraints. The coefficient of Type-II functional divergence (θ_II_) had considerable variation, ranging from −1.83 to 0.60, with about half smaller than 0 (Table 1). Interestingly, the coefficients of both θ_I_ and θ_II_ were significant between PAT1/HAM, SHR/DELLA, SHR/SCR, and SHR/LISCL, suggesting that these GRAS subfamilies, particularly PAT1/HAM, might have experienced both Type-I and Type-II functional divergence. The other paired comparisons between subfamilies with negative θ_II_ value most likely adopted Type-I functional divergence. Additionally, we calculated the posterior probability (*Q*_k_) value to evaluate the possibility of functional divergence between two subfamilies, and considered 0.9 as the *Q*_k_ cutoff in this study. The result showed that the LISCL/DELLA, LISCL/HAM, LISCL/SCR, PAT1/HAM, SHR/LISCL, and SHR/SCR pairs contained 24, 19, 24, 22, 24, or 37 significantly altered aa sites, respectively, by Type-I functional divergence analysis (Table 1). In sum, our findings revealed the significant Type-I functional divergence and some substantially site-specific aa residue changes among GRAS subfamilies.

### 2.3. Conserved Motifs and Gene Structure Analyses

**Figure 2:**
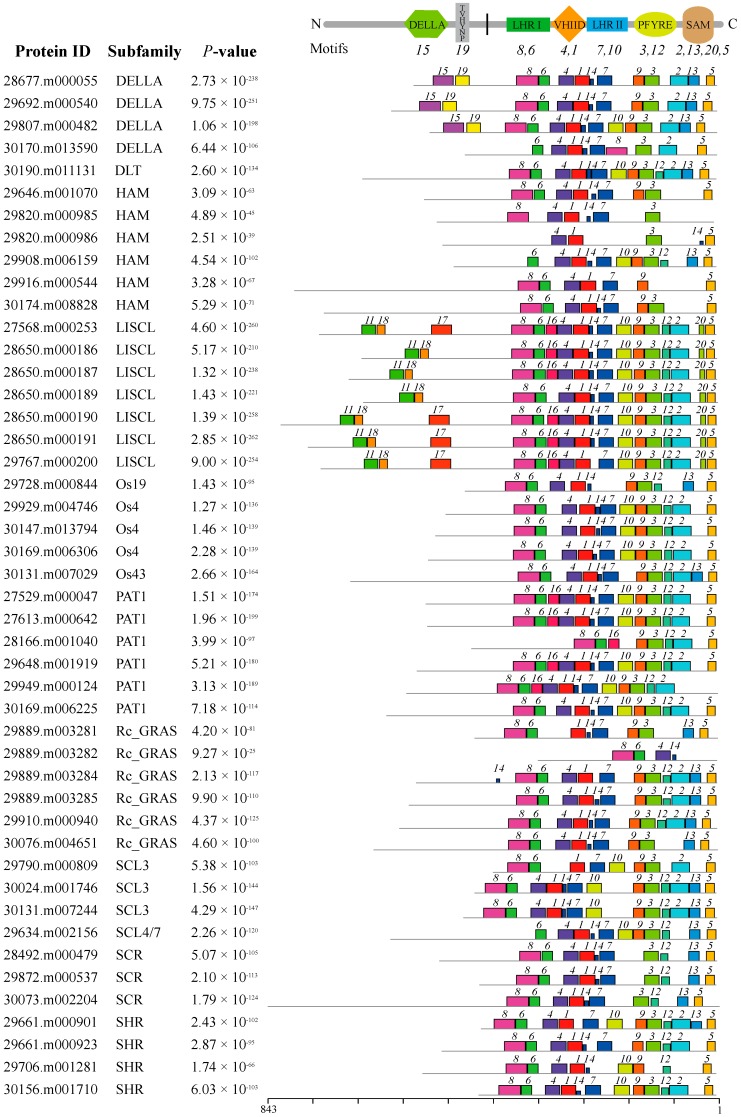
The distribution of conserved motifs within GRAS proteins in castor beans. The different colors indicate the conserved motifs within each protein. The conserved domains within the GRAS protein sequences and corresponding motifs are shown at the top. The scale for protein length is shown at the bottom.

**Figure 3:**
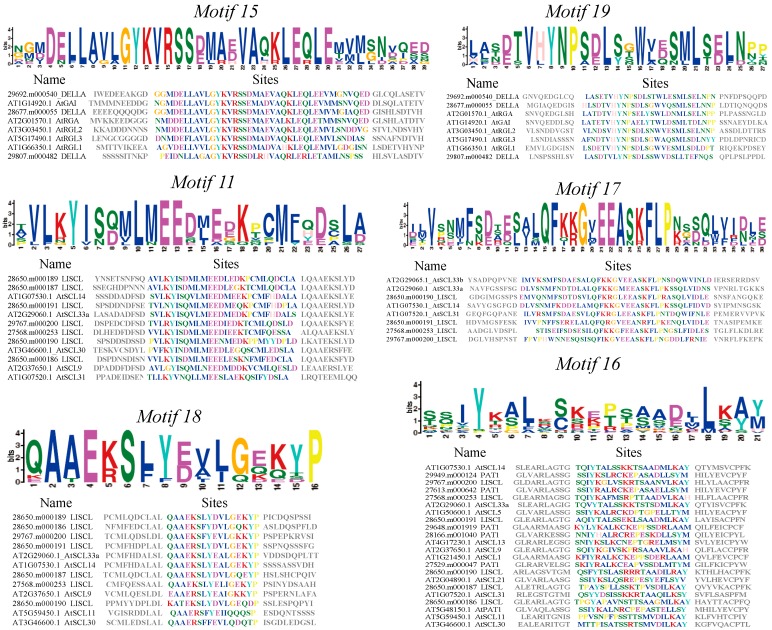
Subfamily-specific motifs nested in the N-terminus region of GRAS proteins in castor beans. The logo represents conserved amino acids sequences between castor beans and *Arabidopsis* included in the motif. Heights of letters in the logo show the frequency of amino acids at that position (**above**). The aligned sequences and conserved amino acids for each motif (colored letters) are shown (**below**).

All GRAS proteins identified in castor beans were subjected to MEME analysis [45] to identify conserved motifs. A total of 20 conserved motifs were found (named Motifs 1–20), and most of motifs displayed similar patterns within the same subfamily (Figure 2). Detailed information for each motif is listed in Supplementary Table S2. Motifs were ordered according to the domain arrangement, with Motifs 8 and 6 corresponding to the LHR I domain, Motifs 4 and 1 corresponding to the VHIID domain, Motifs 7 and 10 corresponding to the LHR II domain, Motifs 3 and 12 corresponding to the PFYRE domain, and Motifs 2, 13, 20, and 5 corresponding to the SAM domain (see Figure 2). Conserved Motif 14 was located between the VHIID and LHR II domains, while Motif 9 was nested between the LHR II and PFYRE domains (see Figure 2). Interestingly, the six other motifs were located outside the GRAS domain regions and nested within specific subfamilies in the phylogenetic tree. For instance, Motifs 15 and 19 were distributed within the DELLA subfamily; Motifs 11, 17, and 18 were within the LISCL subfamily, and Motif 16 was nested in the PAT1 subfamily (see Figure 2 and Figure 3).

**Figure 4:**
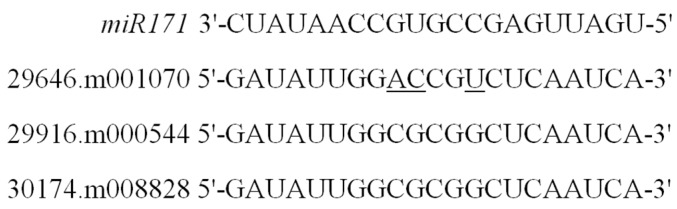
Potential target GRAS members of *Rco-miRNA 171* in castor beans. The underline indicates the non-complementary site to *miR171*.

Based on the sequence alignment between coding sequences and genomic sequences for each GRAS member, exon and intron splicing patterns were identified (Supplementary Figure S3). Most *GRAS* genes (78.3%) had no intron, while seven members contained just one intron, and three members (29648.m001919, 29889.m003282, and 29889.m003284) contained more than one intron. Since recent studies have shown that some GRAS members are regulated by microRNA 171 [29,30,31], we investigated the binding sites targeted by *Rco*-*miRNA 171* from castor beans previously reported [46] for each member of the *GRAS* gene family. It was shown that three genes from the HAM subfamily (29646.m001070, 29916.m000544, and 30174.m008828) were unambiguously complementary to the *Rco*-*miRNA 171* sequence (see Figure 4).

### 2.4. Expression Profiles of GRAS Genes in Different Tissues or Organs

**Figure 5:**
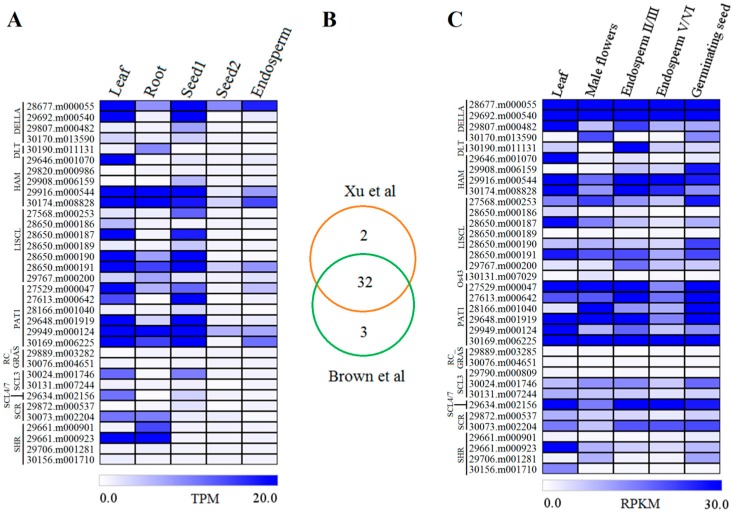
Gene expression profiles of GRAS members in different tissues or organs in castor beans. (**A**) Gene expression data from Xu et al. [47]; (**B**) the overlap of *GRAS* gene expression between Xu et al. [47] and Brown et al. [48]; (**C**) gene expression patterns from Brown et al. [48].

Increasing evidences demonstrate the critical roles of *GRAS* genes in plant developmental processes. To explore the potential functions of these genes in castor beans, we investigated the expression levels of each GRAS member in leaf, root, seed1 (early seed development), seed2 (late seed development), and endosperm using our previous Tag-seq data [47]. Transcripts were detected in at least one of the five tissues for 34 *GRAS* genes, but were not detected in our dataset for the other 12 genes. A total of 31 *GRAS* genes were transcribed in leaf, 25 in root, 27 in seed1, 19 in seed2, and 22 in endosperm (Figure 5A). Expression levels of individual *GRAS* gene varied widely in different tissues. For example, 17 genes (30174.m008828, 29646.m001070, 28650.m000191, 28677.m000055, 29916.m000544, 28650.m000187, 29949.m000124, 29648.m001919, 28650.m000190, 29692.m000540, 30169.m006225, 29661.m000923, 27529.m000047, 27613.m000642, 30024.m001746, 29634.m002156, and 30073.m002204) were highly expressed in leaf, and eight genes (30174.m008828, 29949.m000124, 29916.m000544, 29661.m000923, 28650.m000191, 30169.m006225, 29661.m000901, and 30190.m011131) were highly expressed in root tissue. During seed development, many of the *GRAS* genes (14 members) exhibited a high expression level in early development (seed1), whereas all genes were significantly downregulated later in seed development (seed2). Only six genes (28677.m000055, 30174.m008828, 30169.m006225, 28650.m000191, 29916.m000544, and 29949.m000124) were identified with high expression from endosperm tissues dissected from immature seeds (Figure 5A).

Gene expression analysis of castor bean GRAS members was enhanced by screening Brown et al.’s [48] database (accession number ERA047687) in addition to our library [47]. In total, 35 of 46 *GRAS* genes were detected in at least one library. Furthermore, *GRAS* gene expression was compared between these two databases. The results showed that most genes (32) were detected in both and five with very low expression were identified in only one of the databases. Three genes (30131.m007029, 29889.m003285, and 29790.m000809) were absent in our library, whereas two genes (29820.m000986 and 29889.m003282) were not detected in Brown et al.’s database (Figure 5B). Within the same tissue, GRAS members generally exhibited similar expression patterns (Figure 5C) between databases, consistent with our previous data [47]. Similar to the results observed in seed development, most of the genes with high expression in endosperm II/III were downregulated in the late endosperm stage (endosperm V/VI) (Figure 5C). Moreover, we also identified ten *GRAS* genes that were highly transcribed in male flowers, and 15 genes highly expressed in germinating seeds (Figure 5C). Interestingly, few genes exhibited a tissue-specific expression pattern, and members from Os4, Os19, and Rc_GRAS subfamilies were not detected in any of the tested tissues. Collectively, expression was detected from 38 *GRAS* genes in diverse tissues or organs.

### 2.5. GRAS Gene Responses to Different Stress Treatments

**Figure 6:**
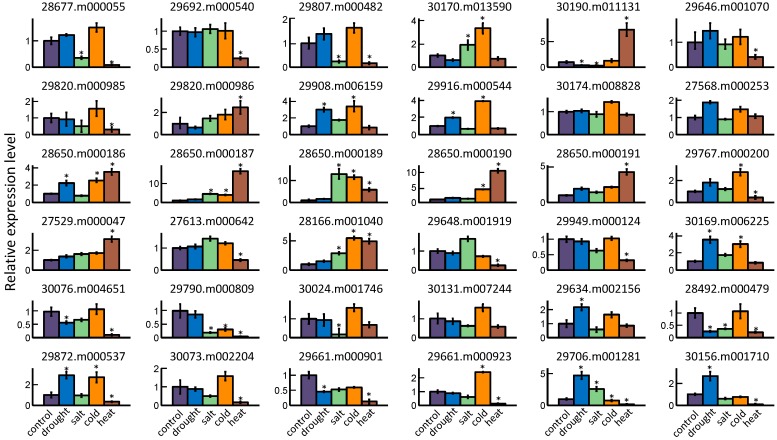
Relative expression levels of 36 *GRAS* genes in different abiotic stress treatments. The two-week-old castor bean seedlings were subjected to various stress treatments including drought, salt, cold, and heat. The columns represent the expression levels of a gene, and the error bars show the standard error with three biological replicates. The expression level of control sample was normalized to 1. The asterisk above the columns indicates a significant change of expression level between stress treatment and control at *p* < 0.05.

The change of expression level of each *GRAS* gene was measured in response to different abiotic stresses. In the current study, four stress treatments including drought, salt, cold, and heat were imposed on castor bean seedlings to elucidate the potential role of these genes under stress. Using quantitative RT-PCR, we detected a total of 36 *GRAS* genes that were responsive to at least one abiotic stress treatment. The remaining members (10 genes) from Os4, Os19, Os43, and Rc_GRAS subfamilies were unable to be detected and showed no response (Figure 6). Increased expression levels were observed in 16 *GRAS* members following drought condition, among which eight genes (29706.m001281, 30169.m006225, 29908.m006159, 29872.m000537, 30156.m001710, 28650.m000186, 29634.m002156, and 29916.m000544) significantly increased their expression at least twofold, while only four genes (28492.m000479, 30190.m011131, 29661.m000901, and 30076.m004651) exhibited obvious downregulation. Salt stress treatment showed an induction of 12 genes, and five genes (28650.m000189, 28650.m000187, 28166.m001040, 29706.m001281, and 30170.m013590) were significantly upregulated more than twofold. The expression levels of six genes (30024.m001746, 29790.m000809, 29807.m000482, 30190.m011131, 28677.m000055, and 28492.m000479) underwent the obvious downregulation during salt treatment. Cold stress treatment affected 14 *GRAS* genes, among which 12 and 2 genes exhibited an obvious increase and decrease, respectively. Genes 28650.m000189 and 29790.m000809 had the greatest change in expression levels following stress. By contrast, nine genes (28650.m000187, 28650.m000190, 30190.m011131, 28650.m000189, 28166.m001040, 28650.m000191, 28650.m000186, 27529.m000047, and 29820.m000986) were substantially upregulated, and 18 genes were markedly downregulated in seedlings subjected to heat stress. For example, the expression levels of two genes (28650.m000187 and 28650.m000190) were elevated more than tenfold under heat treatment relative to that in control plant. In addition, we found that five genes (30190.m011131, 28650.m000187, 28650.m000190, 28650.m000191, and 27529.m000047) could be induced, and two genes (29790.m000809 and 29661.m000901) were repressed in all five stress treatments, implying that these genes might have a potential role in response to various stress treatment.

## 3. Discussion

GRAS transcription factors play pivotal roles in regulating plant growth, development, and stress responses and have been widely investigated in a number of plants [6,9,10,11,12,13,14,15]. In this study, we identified and confirmed the gene structure of 48 putative *GRAS* genes from the current castor bean genome. The number of GRAS members identified in castor beans is higher than in *Arabidopsis* (33 members) [6], comparable to *Jatropha curcas* (48 members) [14], *Prunus mume* (46 members) [11], and Chinese cabbage (46 members) [10], and lower than the number identified in rice (57 members) [6], *Populus* (106 members) [9], and tomato (53 members) [13]. Taking genome size variation into consideration, we examined the density (number/Mb) of *GRAS* genes for each species. The density of *GRAS* genes in the castor bean genome (0.15) is less than *Arabidopsis* (0.24) and *Populus* (0.25), but nearly equal to other species, including rice (0.15), *Jatropha curcas* (0.15), *Prunus mume* (0.16), and Chinese cabbage (0.17). The variation in *GRAS* gene number and density among these species might be related to gene duplication events or the aforementioned genome size.

The GRAS members from castor bean, rice, and *Arabidopsis* were clustered into 14 clades in this study. Interestingly, we found that the Rc_GRAS subfamily only contained the members from castor beans, and rice-specific subfamilies were not detected in *Arabidopsis*. As previously reported, these species-specific subfamilies such as the Rc_GRAS subfamily in castor bean and the Pt20 subfamily in *Populus* [9] were present in many dicotyledons but absent in *Arabidopsis* or in some Poaceae species [14]. These results indicated the loss of some GRAS subfamilies from some plant species during evolution [14]. Moreover, our analysis showed the probable functional divergence and diversification of GRAS evolution among different subfamilies, consistent with reports in other plant species [9,49]. Among the subfamilies, LISCL was the largest subfamily with seven members. The HAM, Rc_GRAS, and PAT1 subfamilies also consisted of multiple members. Interestingly, tandem repeats of GRAS members were observed in these four subfamilies, corresponding with the results from other plant species [49] and indicating that tandem duplication may be a mechanism for expanding GRAS member numbers in these subfamilies.

The distribution of conserved motifs in castor bean GRAS proteins further supports the categorization of the GRAS family. Conserved motifs and amino acid residues were found within the GRAS domain regions that might play an important functional role. For instance, Motif 8 contained a relatively conserved leucine repeat, and Motif 6 contained a putative nuclear localization signal (NLS) located between two arginine residues within the LHR I domain (Supplementary Table S2), a region previously described [6,8]. Within the LHR II region, Motif 10 was present in more than half of GRAS members and contained the conserved LXXLL pattern (Supplementary Table S2) that participates in protein interactions [3,8,50,51]. Residues D (Asp)/E (Gln) have also proven pivotal for protein function within the PFYRE region [3]. In addition, there is evidence that the conserved linker sequences, located between LRII and PFYRE domains, as was observed in conserved Motifs 9, are important for the LYHLL motif (Motif 10)—in particular, flexibility and substrate binding [8]. Outside the GRAS domains, some conserved motifs including Motifs 11 and 15–19 (Figure 3) had subfamily-specific distribution patterns that may endow the GRAS members with specific functions. Similar to reports from other plants, most GRAS members from castor beans lacked an intron (78.3%), which may be a reflection of their ancient origin from prokaryotic genomes of bacteria and horizontal gene transfer [52]. In addition, studies showed that the expression level of some GRAS members could be regulated by microRNA 171 during plant development [29,30,31]. In this study, three *GRAS* genes within the HAM subfamily contained potential target sites for binding *Rco*-*miRNA 171*, suggesting a potential regulatory mechanism for these genes in castor beans.

The expression profiles demonstrated that a total of 38 GRAS members could be detected in castor beans. These members exhibited a broad range of expression patterns among different tissues. Interestingly, eight GRAS members from four subfamilies (Os4, Os19, Os43, and Rc_GRAS) were not expressed in any tissue or organ, suggesting the loss of their underlying function during evolution. By contrast, a high expression of GRAS members in some tissues pointed to their functional roles in development. For example, two genes (29661.m000923 and 30073.m002204) were highly expressed in root tip tissue, which is consistent with the expression of their homologous genes AtSHR and AtSCR, participating in root development in *Arabidopsis* [1,16,17]. Three genes in the PAT1 subfamily (29949.m000124, 27529.m000047, and 27613.m000642) were highly transcribed in leaf tissue; their *Arabidopsis* homologs (AtPAT1, AtSCL13, and AtSCL21) are functionally involved in phytochrome signal transduction [21,22]. Four members of the LISCL subfamily (27568.m000253, 28650.m000187, 28650.m000190, and 28650.m000191) were strongly expressed in male flowers, which is in agreement with research showing LISCL protein serves as a transcriptional activator in the microsporogenesis of the lily anther [53]. Interestingly, we identified two DELLA genes (28677.m000055 and 9692.m000540) that are present and expressed in all tested tissues, consistent with their role as the main signaling hub in the control of a variety of growth and developmental processes [54,55]. It is worth noting that most GRAS members were downregulated during the transition from early to mature seed development, suggesting that these genes are likely related to early seed development.

Many studies have described the potential regulatory roles of *GRAS* genes under various abiotic stresses [55]. In our study, we found that most of castor bean *GRAS* genes (about 78.2%) can be affected by different stress conditions. For instance, the gene 27529.m000047 could be induced under different stress treatments and its homolog in *Arabidopsis*, AtSCL3, has been found to respond to various abiotic stresses and phytohormones [21]. In another example, over-expression of SCL7 from poplar improved salt and drought tolerance in transgenic *Arabidopsis* plants [35]. Gene 29634.m002156 from castor beans was significantly induced by drought treatment and encodes an identical protein sequence as *Arabidopsis* genes AtSCL7 and AtSCL4. DELLA proteins have shown the functional role in regulating stress tolerance in plants [56]. The DELLA member 28677.m000055 was upregulated under cold condition but was downregulated under salt and heat treatment; expression of the 28677.m000540 gene was also repressed following heat stress. In addition, we observed that several castor bean *GRAS* genes had dramatic changes in expression level under more than one abiotic stress treatments, indicating a coordinated response of these members. In particular, the LISCL and PAT1 subfamily showed response to four abiotic stresses, suggesting a wide role in tolerance. Taken together, these results show the potential for castor bean GRAS proteins to function under adverse growth environments.

## 4. Experimental Section

### 4.1. Identification of GRAS Gene Family in Castor Beans

An extensive search was carried out to detect all members of the GRAS transcription factors based on the castor bean genome database (downloaded from the website http://castorbean.jcvi.org/index.php). All *Arabidopsis* and rice GRAS proteins (described by Tian et al. [6]) were downloaded and respectively designed as query sequences against the castor bean protein database by running the ncbi-blast-2.2.24+ software (version 2.2.24+, NCBI, 2011, downloaded from ftp://ftp.ncbi.nih.gov/blast/executables/blast+/2.2.24/). Subsequently, all hit protein sequences were extracted using a custom Perl script based on their gene/protein ID number, and these sequences were then confirmed by the presence of the characterized GRAS domain in the candidate sequences by SMART tools [40]. Further, the CDS (coding sequence) and gene sequences of candidate members of GRAS family were obtained from the website (https://phytozome.jgi.doe.gov/pz/portal.html) for further analyses.

### 4.2. Phylogenetic and Evolutionary Analyses of the GRAS Gene Family

All GRAS protein sequences from *Arabidopsis*, rice, and castor beans were subjected to Clustal W software [44]. Subsequently, an unrooted phylogenetic tree was constructed using neighbor-joining criteria in MEGA 5.0 with 10,000 bootstrap replicates [57]. GRAS members in castor beans were further divided into different subfamilies based on the homologs in *Arabidopsis* and rice. The functional divergence between any two subfamilies was estimated using the DIVERGE v3.0 package [43]. In addition, conserved motif analysis in castor bean GRAS proteins was performed by the MEME tools with 20 motif numbers. The exon–intron patterns were also examined by the online GSDS 2.0 tools (Gene Structure Display Server [58]) based on the CDS and corresponding to genic sequences.

### 4.3. Gene Expression Profiles among Different Organs

The global expression profiles of *GRAS* genes identified in castor beans were analyzed based on our previous Tag-seq data [47]. In brief, all clean reads from leaf, root, seed1 (15 days after pollination), seed2 (35 days after pollination), and endosperm tissues (40 days after pollination) described earlier were mapped to the castor bean genome (http://castorbean.jcvi.org/index.php) using SOAP2 software [59]. The reads mapping to *GRAS* gene regions were extracted by a custom Perl script and the transcription level of each gene in the different tissues was normalized to the number of transcripts per million clean (TPM). Finally, the expression levels of all *GRAS* genes were visualized by the Multi-Experiment View (MEV) cluster software.

### 4.4. Abiotic Stress Treatments in Castor Bean Seedlings

Castor bean seeds were surface sterilized and germinated in MS medium at 28 °C for two weeks. Afterward, the seedlings were transferred to various conditions for stress treatment. Under drought condition, the seedlings were placed on the filter paper for 12 h at 28 °C. For the salt stress treatment, the seedlings were transferred into MS medium containing 300 mM NaCl for 12 h. For cold stress treatment, the seedlings were kept in 4 °C for 12 h, while for heat stress treatment the seedlings were cultured in MS medium at 50 °C for 12 h.

### 4.5. mRNA Isolation and qRT-PCR

Total mRNA was extracted from the whole seedlings of each treatment using RNAprep Pure Plant Kit (Tiangen, Beijing, China). The mRNA with DNA enzyme digestion was used for first-strand cDNA synthesis using PrimeScript™ RT reagent Kit (TaKaRa, Dalian, China), and quantitative RT-PCR amplification for each stress treatment was then carried out with three independent biological replications in Bio-Rad CFX Manager system (BIO-RAD, Berkeley, CA, USA). The qRT-PCR conditions used were as follows: precycling steps of 95 °C for 2 min and then followed by 40 cycles of 95 °C for 30 s, 56 °C for 30 s, and 72 °C for 30 s. The *RcACTIN2* gene was used as an internal reference to normalize the relative expression level of all genes. The primers used in this study are listed in Supplementary Table S3.

## 5. Conclusions

In conclusion, the current study identified and characterized 46 *GRAS* gene family members from the castor bean genome. Further, the phylogenetic relationships, functional divergence between subfamilies, conserved motifs, expression profiles across different tissues, and abiotic stress responses were investigated for these genes in detail. Our results provide comprehensive information towards better understanding the molecular basis and impact of *GRAS* gene families on the growth and development of castor beans. In addition, these results will aid in efforts to identify the mechanisms that allow this crop to grow in varied environmental conditions.

## Figures and Tables

**Table 1:** Functional divergence between subfamilies of GRAS proteins.

Comparison	θ_II_ (s.e.)	θ_I_ (s.e.)	LRT θ_I_	Q_k_ > 0.9
PAT1/SHR	−0.14 (0.64)	0.53 (0.12)	19.29	3
PAT1/LISCL	−0.09 (0.52)	0.57 (0.12)	21.59	4
PAT1/SCL3	−0.83 (0.88)	0.42 (0.18)	5.50	0
PAT1/DELLA	−0.16 (0.50)	0.40 (0.19)	4.38	0
PAT1/SCR	0.42 (0.38)	0.70 (0.15)	22.69	6
PAT1/HAM	0.53 (0.47)	0.87 (0.12)	53.81	22
PAT1/GRAS	0.49 (0.36)	0.70 (0.17)	17.09	5
SHR/LISCL	0.52 (0.28)	0.87 (0.13)	42.95	24
SHR/SCL3	−0.31 (0.91)	0.46 (0.18)	6.76	1
SHR/DELLA	0.60 (0.20)	0.83 (0.20)	16.88	9
SHR/SCR	0.42 (0.58)	0.98 (0.25)	15.70	37
SHR/HAM	−0.28 (0.89)	0.61 (0.14)	17.82	5
SHR/GRAS	0.52 (0.27)	0.61 (0.15)	17.17	2
LISCL/SCL3	−0.70 (0.73)	0.42 (0.16)	7.43	0
LISCL/DELLA	−0.28 (0.45)	0.86 (0.10)	70.47	24
LISCL/SCR	0.35 (0.45)	0.86 (0.14)	39.74	24
LISCL/HAM	0.25 (0.52)	0.82 (0.10)	63.63	19
LISCL/GRAS	0.18 (0.39)	0.50 (0.10)	25.85	4
SCL3/DELLA	−0.79 (0.76)	0.33 (0.21)	2.54	0
SCL3/SCR	−1.83 (1.36)	0.62 (0.20)	9.98	2
SCL3/HAM	−1.18 (1.22)	0.68 (0.14)	22.61	5
SCL3/GRAS	−0.15 (0.65)	0.42 (0.19)	4.73	0
DELLA/SCR	0.37 (0.44)	0.74 (0.22)	11.75	3
DELLA/HAM	0.47 (0.33)	0.74 (0.14)	29.55	9
DELLA/GRAS	0.39 (0.29)	0.58 (0.16)	12.54	2
SCR/HAM	−0.22 (0.85)	0.46 (0.12)	13.96	3
SCR/GRAS	−0.38 (0.67)	0.58 (0.18)	10.66	1
HAM/GRAS	0.27 (0.50)	0.55 (0.10)	27.61	5

θ_I_ and θ_II_: the coefficients of Type I and Type II functional divergence. LRT: likelihood ratio test statistics. *Q*_k_: posterior probability.

## Supplementary Materials

Supplementary materials can be found at http://www.mdpi.com/1422-0067/17/7/1004/s1.

## Abbreviations

GAs: gibberellins
phyA/B: phytochrome A/B
aa: amino acid residues
ORF: open reading frame
CDS: coding Sequence
