# Supplementary material for: Genome-Wide Identification, Evolutionary Analysis, and Stress Responses of the GRAS Gene Family in Castor Beans
Source: Int J Mol Sci. 2016 Jun 24;17(7):1004. doi: 10.3390/ijms17071004 (PMC4964380; doi:10.3390/ijms17071004)
Supplement: Supplementary file 1 [file ijms-17-01004-s001.pdf]

Supplementary Materials: Genome-Wide Identification, Evolutionary Analysis and Stress Responses of *GRAS* Gene Family in Castor Bean

Wei Xu, Zexi Chen, Naeem Ahmed, Bing Han, Qinghua Cui and Aizhong Liu

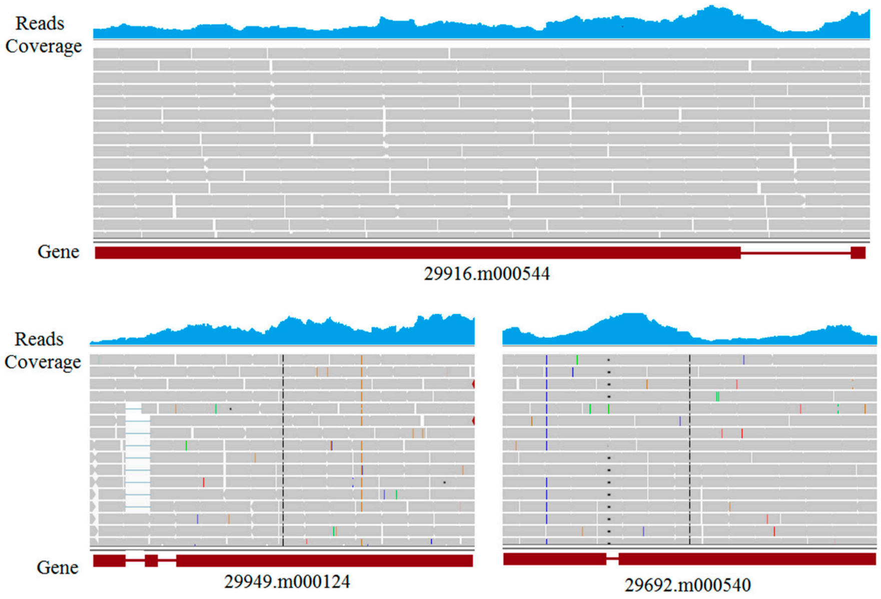

Figure S1. Visualization of the reads coverage on genic regions of three predicted *GRAS* genes.

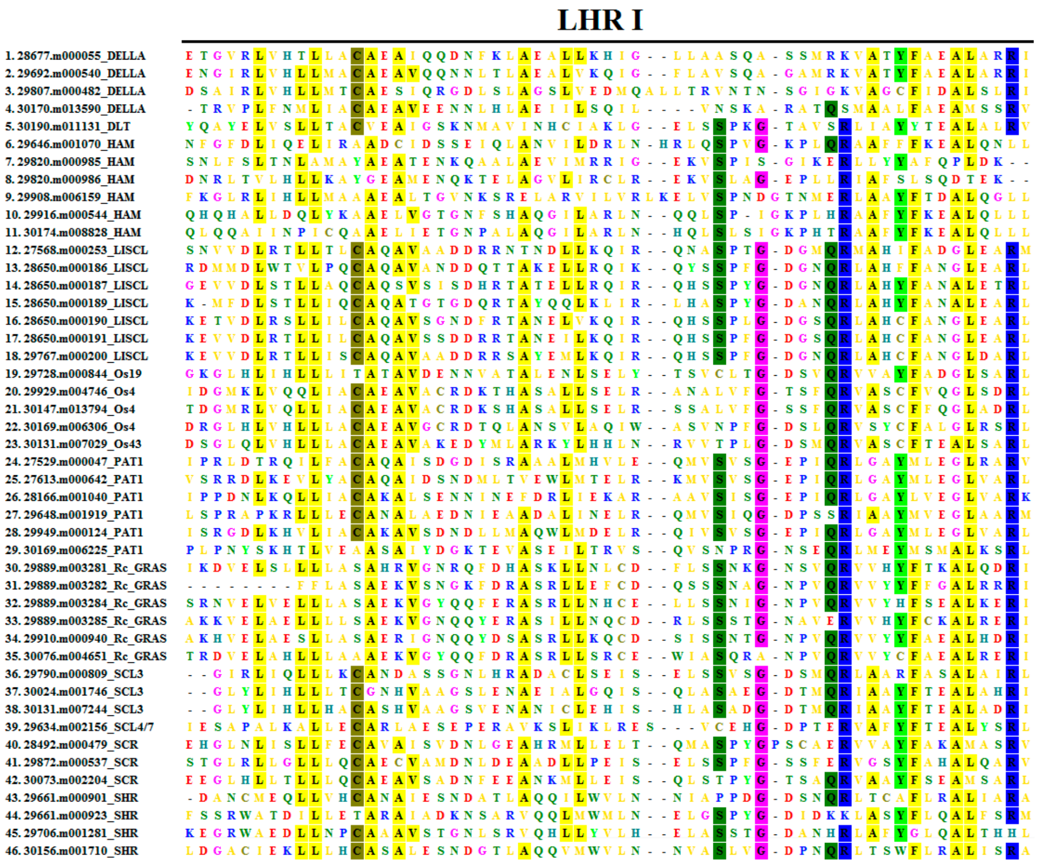

Figure S2. *Cont.*

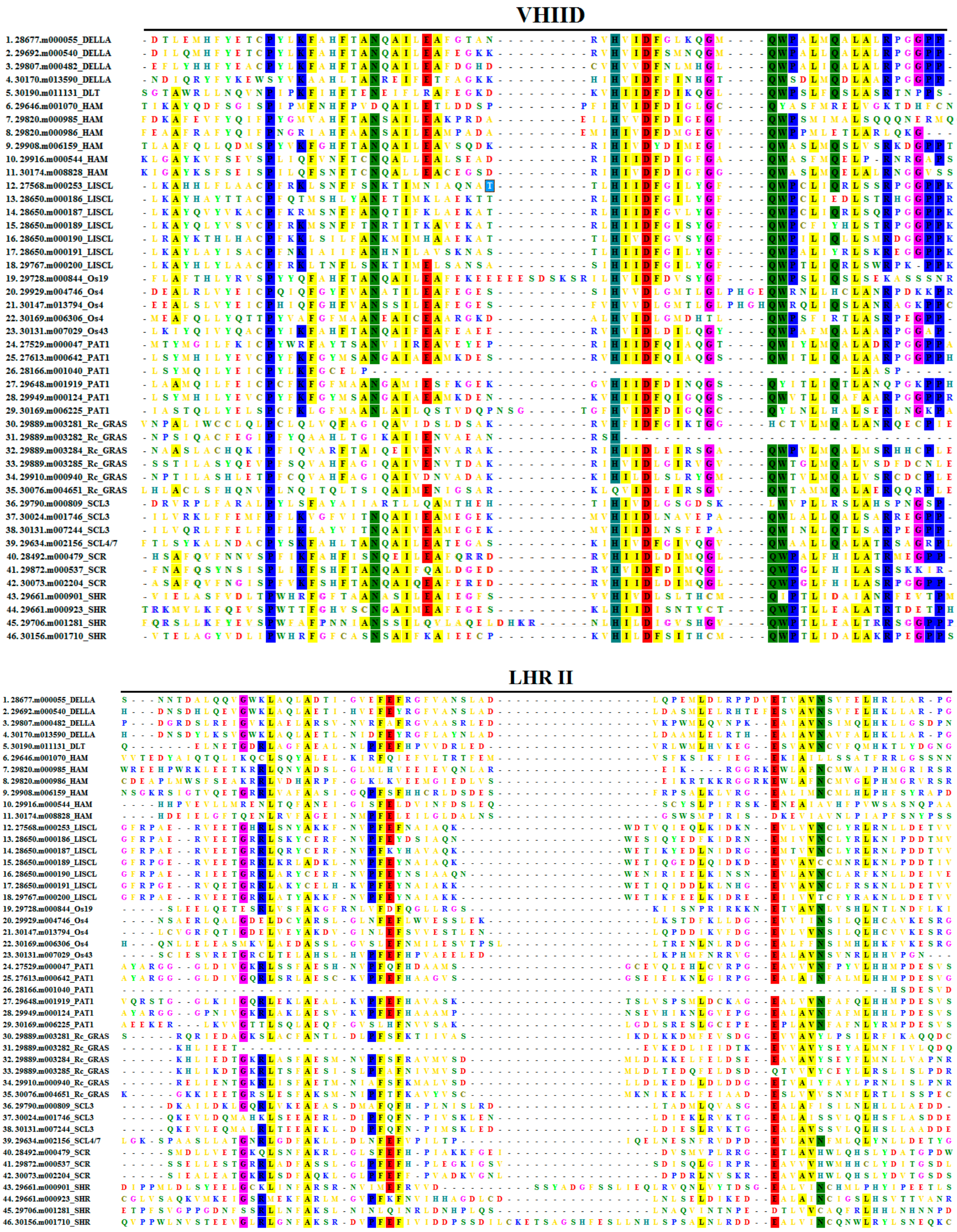

Figure S2. *Cont.*

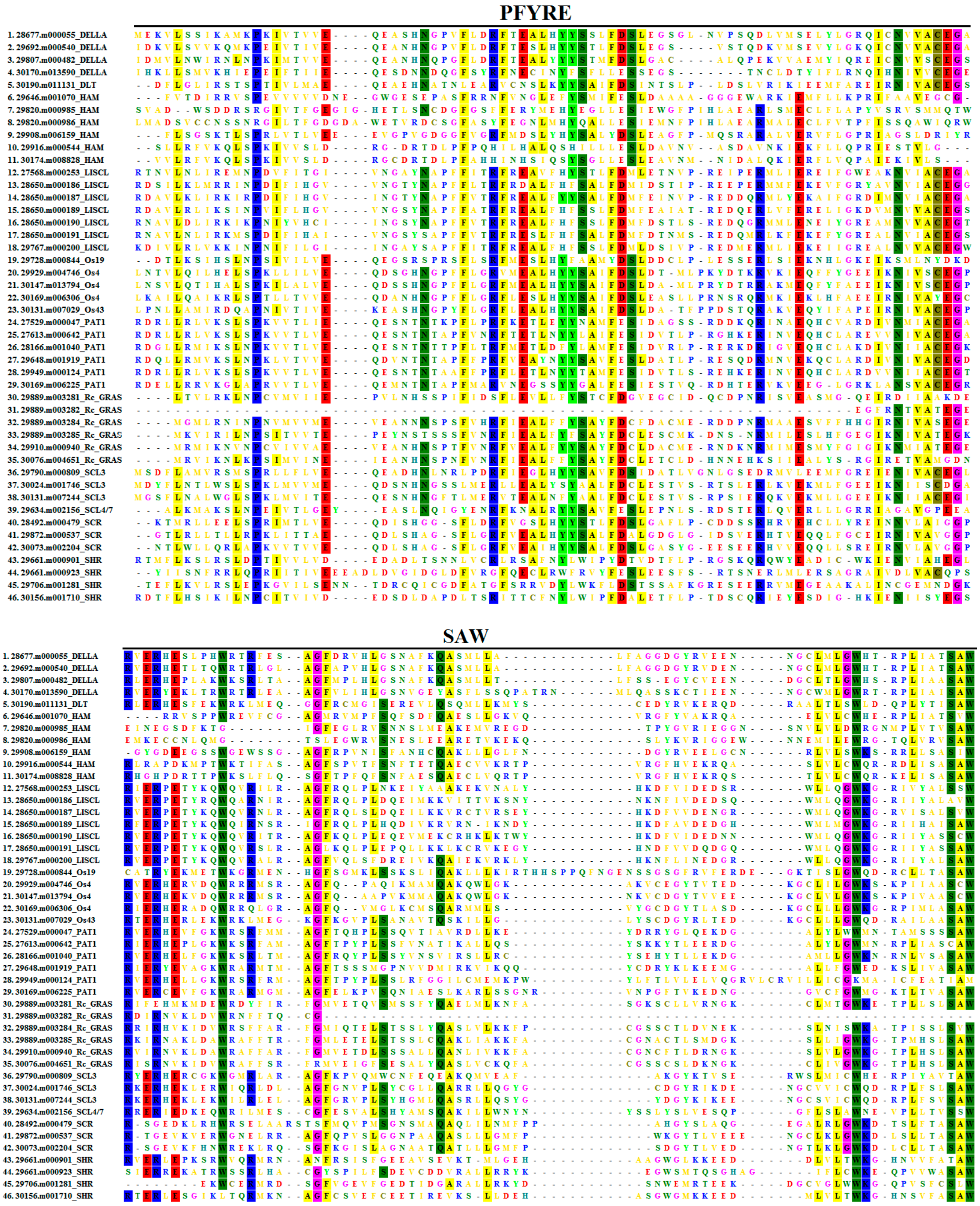

Figure S2. Multiple sequences alignment of amino acid of the conserved domains including LHR I, VHIID, LHR II, PFIYE and SAM in castor bean.

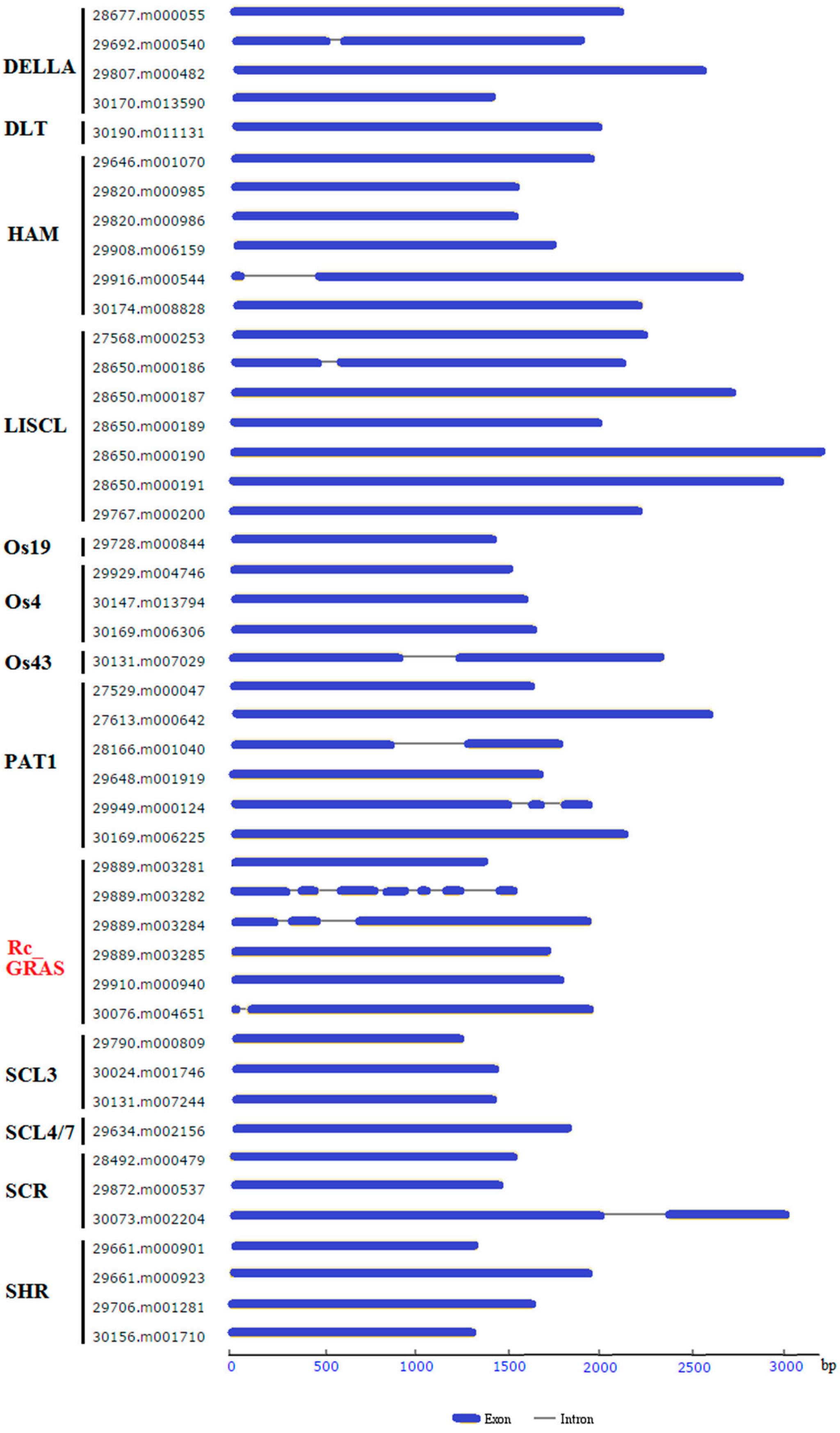

Figure S3. Gene structures of GRAS members in castor bean.

Table S1. The information of 48 GRAS transcription factors identified in castor bean genome.

Gene Model	Scaffold	Gene Start	Gene End	Orientation	Domain Number	*E*-Value (Pfam)	CDS Length	Protein Length	Molecular Weight/Da	Theoretical pI	Reads Coverage (>10)	Homologs in *Arabidopsis*
27529.m000047	27529	20956	22584	+	1	1.00E-123	1629	542	61122.84	5.72	yes	AT4G17230.1
27568.m000253	27568	19777	22002	+	1	1.00E-120	2226	741	83116.30	5.37	yes	AT1G07530.1
27613.m000642	27613	218157	220737	-	1	2.70E-137	1617	538	60047.72	5.50	yes	AT1G50600.1
28166.m001040	28166	23949	25728	-	2	6.40E-17	1380	459	51523.05	6.25	yes	AT5G48150.1
28492.m000479	28492	123817	125376	-	1	7.10E-104	1560	519	58016.83	5.92	no	AT3G54220.1
28650.m000186	28650	27256	29354	-	1	2.30E-108	1992	663	75257.66	5.92	no	AT1G07530.1
28650.m000187	28650	31218	33926	-	1	2.80E-118	2067	688	78173.80	6.32	yes	AT1G07530.1
28650.m000189	28650	41918	43906	-	1	1.80E-112	1989	662	75340.73	6.16	no	AT1G07530.1
28650.m000190	28650	48435	51623	-	1	1.60E-113	2448	815	90986.60	5.17	yes	AT1G07530.1
28650.m000191	28650	57247	60227	-	1	6.90E-110	2295	764	85580.05	5.28	yes	AT1G07530.1
28677.m000055	28677	3350	5464	+	1	3.40E-36	1704	567	62550.40	5.14	yes	AT3G03450.1
28966.m000535	28966	125327	126678	-	1	4.10E-15	483	160	17305.22	5.88	no	AT1G55580.1
29634.m002156	29634	607639	609465	+	1	7.20E-109	1827	608	66887.21	4.96	yes	AT5G66770.1
29646.m001070	29646	113881	115836	-	1	4.80E-87	1620	539	60899.08	6.03	yes	AT4G36710.1
29648.m001919	29648	47547	49226	-	1	5.10E-136	1680	559	62597.52	4.89	yes	AT1G21450.1
29661.m000901	29661	20973	22295	-	1	1.60E-89	1323	440	49288.28	5.58	yes	AT3G49950.1
29661.m000923	29661	255682	257645	+	1	7.40E-104	1539	512	58194.98	5.87	yes	AT4G37650.1
29692.m000540*	29692	243910	245798	+	1	2.80E-37	1830	609	67287.91	5.73	yes	AT1G14920.1
29706.m001281	29706	78273	79919	-	1	4.20E-78	1647	548	60960.68	5.85	yes	AT3G13840.1
29728.m000844	29728	310370	311785	-	1	4.20E-89	1416	471	53630.99	6.17	no	AT3G54220.1
29767.m000200	29767	26535	28757	+	1	2.80E-112	2223	740	83933.94	8.40	yes	AT2G37650.1
29790.m000809	29790	48786	50036	-	1	4.30E-99	1251	416	46776.87	6.40	yes	AT1G50420.1
29807.m000482	29807	100421	102935	-	1	5.90E-32	1611	536	58616.68	5.27	yes	AT2G01570.1
29820.m000985	29820	38524	40077	-	1	2.90E-41	1554	517	59681.48	4.82	no	AT4G08250.1
29820.m000986	29820	44796	46325	+	1	8.90E-38	1530	509	57484.06	4.83	no	AT5G66770.1
29872.m000537	29872	117231	118693	-	1	8.40E-114	1329	442	48239.49	5.45	yes	AT5G41920.1
29889.m003281	29889	213307	214671	-	1	2.00E-72	1365	454	51417.81	8.39	no	AT3G03450.1
29889.m003282	29889	216102	217643	-	1	2.30E-10	1008	335	38304.95	5.10	no	AT3G03450.1
29889.m003284	29889	221800	223738	+	1	5.20E-87	1689	562	64012.76	5.50	no	AT1G66350.1
29889.m003285	29889	225519	227249	+	1	1.10E-82	1731	576	64817.84	5.34	no	AT5G17490.1
29908.m006159	29908	273013	274753	+	1	2.30E-99	1476	491	54282.82	5.64	yes	AT4G08250.1
29910.m000940	29910	353874	355658	-	1	3.40E-92	1785	594	67317.41	5.09	no	AT1G66350.1
29916.m000544*	29916	241112	243860	-	1	5.40E-94	2364	787	86369.68	6.32	yes	AT4G00150.1
29929.m004746	29929	1419600	1421123	+	1	2.00E-106	1524	507	56534.09	6.86	no	AT1G50420.1
29949.m000124*	29949	53044	54985	-	1	4.30E-123	1749	582	65343.70	6.30	yes	AT5G48150.1
29957.m001404	29957	285159	285539	-	1	4.60E-09	267	88	10096.76	10.21	no	AT2G01570.1
30024.m001746	30024	1148008	1149444	-	1	8.80E-138	1356	451	50679.56	6.38	yes	AT1G50420.1

Table S1. *Cont.*

Gene Model	Scaffold	Gene Start	Gene End	Orientation	Domain Number	*E*-Value (Pfam)	CDS Length	Protein Length	Molecular Weight/Da	Theoretical pI	Reads Coverage (>10)	Homologs in *Arabidopsis*
30073.m002204	30073	100179	103200	+	1	1.40E-124	2532	843	90820.90	6.11	yes	AT3G54220.1
30076.m004651	30076	1394867	1396826	+	1	1.00E-81	1929	642	73135.35	6.36	no	AT3G03450.1
30131.m007029	30131	1098698	1101046	-	1	2.10E-133	2061	686	75615.20	5.70	no	AT3G54220.1
30131.m007244	30131	2435990	2437414	-	1	2.30E-135	1425	474	53756.93	5.93	yes	AT1G50420.1
30147.m013794	30147	2621522	2623102	+	1	6.50E-111	1581	526	58433.45	5.78	no	AT1G66350.1
30156.m001710	30156	550167	551501	-	1	2.60E-86	1335	444	49717.52	5.13	no	AT3G49950.1
30169.m006225	30169	2156081	2158208	-	1	5.70E-111	1848	615	67440.71	6.16	yes	AT5G52510.1
30169.m006306	30169	395846	397483	+	1	1.30E-109	1638	545	61000.11	4.99	no	AT1G50420.1
30170.m013590	30170	584630	586045	-	1	2.80E-92	1416	471	53788.61	5.68	no	AT2G01570.1
30174.m008828	30174	547369	549570	-	1	2.20E-95	2202	733	80414.81	5.73	yes	AT4G00150.1
30190.m011131	30190	2053590	2055578	-	1	1.20E-127	1989	662	73717.04	5.70	yes	AT1G63100.1

Table S2. Summary of conserved motifs identified from the GRAS proteins in castor bean.

Motif 1

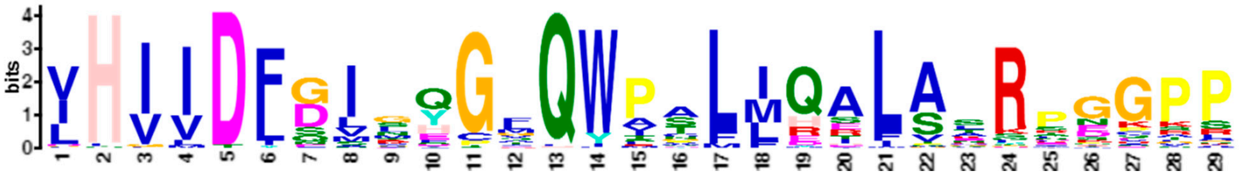

2.	27568.m000253_LISCL	475	4.85e-31	**IMNIAQNATT**	**LHIIDFGILYGFQWPCLIQRLSSRPGGPP**	**KLRITGIDFP**
1.	28677.m000055_DELLA	301	4.85e-31	**ILEAFGTANR**	**VHVIDFGLKQGMQWPALMQALALRPGGPP**	**AFRLTGIGPP**
25.	27613.m000642_PAT1	276	2.20e-29	**IAEAMKDESR**	**VHIIDFQIAQGSQWITLIQALAARPGGPP**	**HVRLTGIDDS**
2.	29692.m000540_DELLA	341	2.20e-29	**ILEAFEGKKR**	**VHVIDFSMNQGMQWPALLQALALRPGGPP**	**AFRLTGIGPP**
28.	29949.m000124_PAT1	285	4.44e-29	**IAEAMKDENK**	**VHIIDFQIGQGSQWVTLIQAFAARPGGPP**	**RIRITGIDDS**
14.	28650.m000187_LISCL	421	6.28e-29	**IFKLAEKATR**	**LHIIDFGVLYGFQWPCLIQRLSQRPGGPP**	**KLRITGIELP**
42.	30073.m002204_SCR	563	2.04e-28	**IQEAFEREDR**	**VHIIDLDIMQGLQWPGLFHILASRPGGPP**	**YVRLTGLGTS**
3.	29807.m000482_DELLA	255	3.34e-28	**ILEAFDGHDC**	**VHVVDFNLMHGLQWPALIQALALRPGGPP**	**LLRLTGIGPP**
15.	28650.m000189_LISCL	396	6.36e-28	**ITKAVEKATR**	**LHIIDFGISYGFQWPCFIYHLSTRPGGPP**	**KVRITGIDYP**
24.	27529.m000047_PAT1	280	8.73e-28	**IREAVEYEPR**	**IHIIDFQIAQGTQWIYLMQALADRPGGPP**	**AIRITGVDDP**
23.	30131.m007029_Os43	434	2.22e-27	**IFEAFEAEER**	**VHVIDLDILQGYQWPAFMQALAARPGGAP**	**FLRITGVGSC**
13.	28650.m000186_LISCL	396	2.22e-27	**IMKLAEKTTR**	**LHIIDFGILYGFQWPCLIEDLSTRHGGPP**	**RLHITGIEFP**

Table S2. *Cont.*

17.	28650.m000191_LISCL	499	4.07e-27	**ILAVSKNAST**	**LHIIDFGILYGFQWPALIYRLSKREGGPP**	**KLRITGIELP**
40.	28492.m000479_SCR	262	6.37e-27	**ILEAFQRRDR**	**VHIIDLDIMQGLQWPALFHILATRMEGPP**	**HIRMTGMGTS**
46.	30156.m001710_SHR	155	2.36e-26	**IFKAIEECPK**	**VHILDFSITHCMQWPTLIDALAKRPEGPP**	**SLRITVPFTR**
18.	29767.m000200_LISCL	476	3.61e-26	**IMELSANSAS**	**IHIIDFGILYGFQWPTLIQRLSWRPKPPK**	**VRITGIDFPQ**
5.	30190.m011131_DLT	391	3.22e-25	**FLRAFEGKDK**	**VHIIDFDIKQGLQWPSLFQSLASRTNPPS**	**HVRITGIGES**
4.	30170.m013590_DELLA	192	6.21e-25	**IFETFAGKKH**	**IHVIDFFINHGTQWSDLMQDLAARPGGPP**	**TIRISGIGFP**
27.	29648.m001919_PAT1	296	1.04e-24	**MIESFKGEKG**	**VHIIDFDINQGSQYITLIQTLANQPGKPP**	**HLRLTGIDDP**
39.	29634.m002156_SCL4/7	344	1.34e-24	**ILEATEGASK**	**IHIVDFGIVQGVQWAALLQALATRSAGRP**	**LSIRISGIPA**
16.	28650.m000190_LISCL	549	1.73e-24	**IMHAAEKATT**	**LHIVDFGVSYGFQWPILIQLLSMRDGGPP**	**KLRITGIELP**
9.	29908.m006159_HAM	220	6.79e-24	**ILEAVSQDKR**	**IHIVDYDIMEGIQWASLMQSLVSRKDGPP**	**TPHLRITALS**
11.	30174.m008828_HAM	481	8.66e-24	**LLEACEGSDR**	**IHIVDFDIGFGGQWASLMQELALRNGGVS**	**SLKITAFVSP**
41.	29872.m000537_SCR	188	2.24e-22	**IFQALDGEDR**	**VHVIDFDIMQGLQWPGLFHILASRSKKIR**	**SMRITGFGSS**
45.	29706.m001281_SHR	276	4.35e-22	**LAQELDHKRN**	**LHILDIGVSHGVQWPTLLEALTRRSGGPP**	**PLVRITVITA**
22.	30169.m006306_Os4	280	4.85e-22	**ICEAARGKDA**	**LHVIDLGMDHTLQWPSFIRTLASRPEGPP**	**KVRITGLIND**
29.	30169.m006225_PAT1	352	6.84e-21	**TVDQPNSGTG**	**FHVIDFDIGQGCQYLNLLHALSERLNGKP**	**ATVKITAVAD**
32.	29889.m003284_Rc_GRAS	303	7.57e-21	**IVENVARAKR**	**IHIIDLEIRSGAQWPVLMQALMSRHHCPL**	**ELLKISAIGT**
34.	29910.m000940_Rc_GRAS	325	1.39e-20	**IVDNVADAKK**	**IHILDLSLRYGMQWTVLMQALVSRCDCPL**	**EHLKITAIGT**
37.	30024.m001746_SCL3	136	2.08e-20	**IIEAMEGEKM**	**VHIIDLNAVEPAQWLALLQALSARREGPP**	**HLRITGIHQQ**
38.	30131.m007244_SCL3	156	2.30e-20	**IVEAMEGEKM**	**VHIIDLNSFEPAQWINLLQTLSARPEGPP**	**HLRITGIHEQ**
10.	29916.m000544_HAM	535	6.14e-20	**LLEALSEADR**	**IHIIDFDIGFGAQWASFMQELPRNRGAPS**	**LKITAFASPS**
44.	29661.m000923_SHR	235	8.21e-20	**IMEAFEGESK**	**LHIIDISNTYCTQWPTLLEALATRTDETP**	**HLRLTTIVAS**
19.	29728.m000844_Os19	198	3.11e-19	**EEESDSKSRI**	**LHVIDFDVSYGFQWPSLIQSLSEKASSSN**	**RISLRITGLG**
30.	29889.m003281_Rc_GRAS	180	1.36e-18	**VIDSLDSAKR**	**VHFIDFGIKTGGHCTVLMQALANRQECPI**	**ELLKISGVGV**
8.	29820.m000986_HAM	235	1.78e-18	**LEAMPADAEM**	**IHIVDFDMGEGVQWPPMLETLARLQKGVR**	**LTAIKWEQEN**
7.	29820.m000985_HAM	238	2.54e-18	**LEAKPRDAEI**	**LHVVDFDIGEGIQWPSMIMALSQQQNERM**	**QQHGYNQRHL**
43.	29661.m000901_SHR	146	2.78e-18	**ILEAIEGFSV**	**VHIVDLSLTHCMQIPTLIDAIANRFEVTP**	**MIKLTVAGCT**
33.	29889.m003285_Rc_GRAS	318	4.73e-18	**IVENVTDAKR**	**IHVIDLGIRVGVQWTGLMQALVSDFDCNL**	**ELLKITAVGT**
35.	30076.m004651_Rc_GRAS	370	8.71e-18	**IMENIGSARK**	**LQVIDLEIRSGVQWTAMMQALAERQQRPL**	**EHLKVTALGL**
6.	29646.m001070_HAM	279	1.51e-16	**LETLDDSPPF**	**IHVIDFDIGLGCQYASFMRELVGKTDHFC**	**NKLTSPVLRI**
21.	30147.m013794_Os4	271	1.19e-15	**FEGESFVHVV**	**DLGMTLGLPHGHQWRQLIQSLANRAGKPP**	**CRLRITAVGL**
36.	29790.m000809_SCL3	144	5.48e-15	**LLQAMTHEHT**	**IHIVDLGSGDSKLWVPLLRSLAHSPNGSP**	**HLKFTCLNTD**
20.	29929.m004746_Os4	251	8.57e-15	**FEGESSIHVV**	**DLGMTLGLPHGEQWRNLLHCLANRPDKKP**	**RCLRITGVGN**

Table S2. *Cont.*

Motif 2

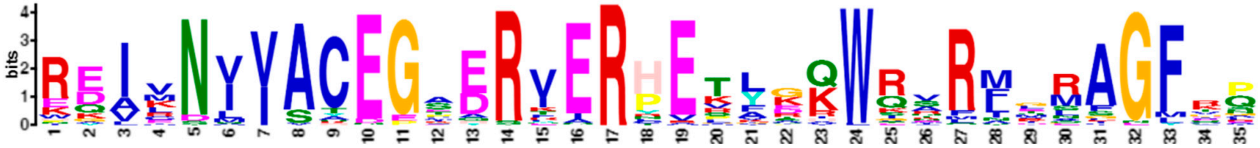

28.	29949.m000124_PAT1	466	1.49e-34	RINVEQHCLA	RDVVNIIACEGTERVERHELLGKWRSRFRMAGFTP	YPLSSLRFGG
12.	27568.m000253_LISCL	656	3.35e-34	RMLIEREIFG	WEAKNVIACEGAERIERPETYKQWQVRILRAGFRQ	LPLNKEIYAA
25.	27613.m000642_PAT1	457	6.32e-34	RINVEQHCLA	REVVNIVACEGAERIERHEPLGKWKSRFAMAGFTP	YPLSSFVNAT
18.	29767.m000200_LISCL	656	6.32e-34	RMLIEKEIIG	REALNVVACEGWERVERPETYKQWQVRALRAGFVQ	LSFDREIVKQ
26.	28166.m001040_PAT1	378	2.51e-33	RIGVEQHCLA	KDIVNIIACEGKERVERHELFGKWKSRLTMAGFRQ	YPLSSYVNSV
2.	29692.m000540_DELLA	516	2.92e-33	DKVMSEVYLG	KQICNVVACEGADRVERHETLTQWRTRLGLAGFAP	VHLGSNAFKQ
27.	29648.m001919_PAT1	477	2.93e-32	RMNVEKQCLA	RDIVNIVACEGDERIERYEVAGKWRARMTMAGFTS	SSMGPNVVDM
13.	28650.m000186_LISCL	577	1.31e-31	RMMFEKEVFG	RYAVNVIACEGGERVERPETYRQWQARNIRAGFRQ	LPLDQEIMKK
17.	28650.m000191_LISCL	680	3.30e-31	RLKFEKEFYG	REALNVIACEGSERVERPETYKQWQVRSLRAGLKQ	LPLEPQLLKK
20.	29929.m004746_Os4	424	4.28e-31	RVKIEQFFYG	EEIKNIVSCEGPARVERHERVDQWRRRMSRAGFQP	AQIKMAMQAK
21.	30147.m013794_Os4	444	1.53e-30	RAKMEQFYFA	EEIKNIVSCEGPARVERHEKVDQWRRRMSRAGFQA	APVKMMAQAK
36.	29790.m000809_SCL3	334	2.22e-30	DRMVLEEMFG	REIENIVACEGLERYERHERCGKWGMRLARAGFKP	VQMWCNFEQE
15.	28650.m000189_LISCL	577	4.11e-30	RLVFERELIG	KDVMNVVACEGSERFERPETYKQWQIRNSRIGFRQ	LPLHQDIVKR
1.	28677.m000055_DELLA	479	9.56e-30	DLVMSELYLG	RQICNVVACEGAHRVERHESLPHWRTRFESAGFDR	VHLGSNAFKQ
3.	29807.m000482_DELLA	432	1.72e-28	EKVVAEMYIQ	REICNVVSCEGSARLERHEPLAKWKSRLTAAGFMP	LHLGSNAFKQ
38.	30131.m007244_SCL3	387	2.15e-28	RQKVEKMLLG	EEIKNIIACEGIERKERHEKLEKWILRLELAGFGR	VPLSYHGMLQ
5.	30190.m011131_DLT	563	1.11e-27	VRIKIEEMFA	REIRNIVACEGSDRLERHESFEKWRKLMEQGGFRC	MGISEREVLQ
29.	30169.m006225_PAT1	531	4.88e-27	ERVKVEEGLG	RKLANSVACEGRDRVERCEVFGKWRARMGMAGFEL	KPVSQNIAES
23.	30131.m007029_Os43	603	1.37e-26	RAKVEQYIFA	PEIRNIVACEGPERTERHERLEKWRKLMEGKGFKG	VPLSANAVTQ
37.	30024.m001746_SCL3	366	1.86e-26	RLKVEKMLFG	EEIKNIISCDGAERKERHEKLERWIQRLDLAGFGN	VPLSYCGLLQ
22.	30169.m006306_Os4	458	2.52e-26	RMKIEKLHFA	EEIRNIVAYEGCDRIERHERADQWRRQLGRAGFQV	MGLKCMSQAR
4.	30170.m013590_DELLA	362	3.95e-25	NCLDTYIFLR	NQIHNIVVCEGEYRVERYEKLTRWRTRLEAAGFVL	IHLGSNVGEY
43.	29661.m000901_SHR	357	1.67e-21	QRQWYEADIC	WKIENVIAHEGLQRVERLEPKSRWVQRMRNANFRS	ISFGEEAVSE
34.	29910.m000940_Rc_GRAS	499	2.83e-20	RMIMESMYFG	IGIKNMIATEGEERVIRNVKLDAWRAFFARFGMVE	TDLSSSALLQ
32.	29889.m003284_Rc_GRAS	477	4.54e-20	RMAAESVFFH	HGIRNIVASEGEERRIRHVKIDVWRSFFARFGMIQ	TELSTSSLYQ
33.	29889.m003285_Rc_GRAS	491	8.47e-20	RMILESLHFG	EGIKNIVATEGKERKIRNAKLDAWRAFFTRFGMLE	TELSTSSLCQ
46.	30156.m001710_SHR	357	1.69e-19	QRIEYESDIG	HKIENIISYEGSQRTERLESGIKLTQRMKNAGFCS	VEFCEETIRE
44.	29661.m000923_SHR	429	4.22e-19	ERLMLERSAG	RAIVDLVACQPSESIERREKATRWSSRLHACGYSP	ILFSDEVCDD

Table S2. *Cont.*

Motif 3

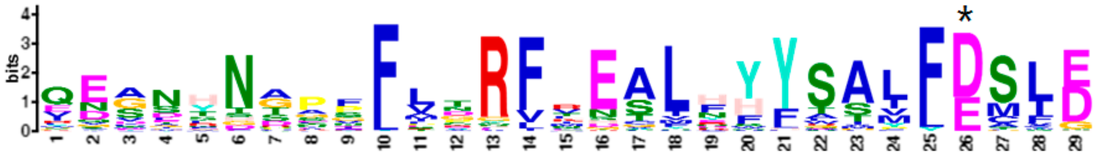

21.	30147.m013794_Os4	396	4.50e-30	LSPKILALVE	QDSSHNGPFFLGRFMEALHYYSAIFDSLD	AMLPRYDTRR
22.	30169.m006306_Os4	409	6.89e-29	LSPTLLTVVE	QDANHNGPFFLGRFLESLHYYSAIFDSLE	ASLLPRNSRQ
2.	29692.m000540_DELLA	471	1.51e-28	MKPEIVTIVE	QEANHNGPVFLDRFTESLHYYSTLFDSLE	GSVSTQDKVM
1.	28677.m000055_DELLA	431	1.51e-28	MKPKIVTVVE	QEASHNGPVFLDRFTEALHYYSSLFDSLE	GSGLNVPSQD
34.	29910.m000940_Rc_GRAS	451	2.68e-28	VNPCVIVVAE	VEANHNSPTFVNRFVEALFYYSAYFDCLD	ACMERNDKNR
20.	29929.m004746_Os4	376	1.41e-27	LSPKLLILVE	QDSGHNGPFFLGRVMEALHYYSAIFDSLD	TMLPKYDTKR
14.	28650.m000187_LISCL	554	2.85e-27	IRPDIFIHGV	INGTYNAPFFVTRFREALFYYSALFDMFE	INVPREDDQR
3.	29807.m000482_DELLA	387	1.32e-26	LNPKIMTVVE	QEANHNQPGFLDRFTEALYYYSTMFDSLG	ACALQPEKVV
35.	30076.m004651_Rc_GRAS	497	2.55e-26	LKPSIMVINE	IEANHNSPNFVNRFIEALFFYSAYFDCLE	TCLDHNNEHK
23.	30131.m007029_Os43	555	4.86e-26	QAPNIVTIVE	KEASHNGPYFLGRFLEALHYYSAIFDSLD	ATFPPDSTQR
16.	28650.m000190_LISCL	683	2.09e-24	IKPNIYVHCI	INGSYNAPFFVTRFREALFHFSSLFDMFD	STLSREDQGR
13.	28650.m000186_LISCL	529	2.40e-24	INPDIFIHGV	VNGTYNAPFFLTRFRDALFHFSALFDMID	STIPREEPER
25.	27613.m000642_PAT1	409	6.38e-24	LSPKVVTLVE	QESNTNTAPFVNRFTETLNYYLAIFESID	VTLPRGHKER
32.	29889.m003284_Rc_GRAS	429	7.32e-24	INPNVMVVME	VEANNNSPSFVHRFIEALFFYSAYFDCFD	ACMERDDPNR
27.	29648.m001919_PAT1	429	7.32e-24	LNPKLVTVVE	QDVNTNTAPFFPRFVEAYNYYSAVFESLD	ATLPRESQDR
15.	28650.m000189_LISCL	529	1.44e-23	INPVIFLHGV	VNGSYNAPFFATRFREALFHFSSLFDMFE	AIATREDQER
12.	27568.m000253_LISCL	608	1.44e-23	MNPDVFITGI	VNGAYNAPFFITRFREAVFHYSTLFDMLE	TNVPREIPER
28.	29949.m000124_PAT1	418	9.01e-23	LSPKVVTLVE	QESNTNTAAFFPRFLETLNYYTAMFESID	VTLSREHKER
24.	27529.m000047_PAT1	413	9.01e-23	LSPKVVTLIE	QESNTNTKPFLPRFKETLEYYNAMFESID	AGSSRDDKQR
17.	28650.m000191_LISCL	632	9.01e-23	MSPDIFIHAI	VNGSYSAPFFVTRFRESLFHFSALFDMFD	TNMSREDQMR
26.	28166.m001040_PAT1	330	1.92e-22	LNPKVVTLVE	QESNTNTTPFLTRFMETLDFYLAMFESID	VRLPRERKDR
18.	29767.m000200_LISCL	608	6.54e-22	INPNIFILGI	INGAYSAPFFITRFREALFHFSSLFDMLD	SIVPREDMER
33.	29889.m003285_Rc_GRAS	444	7.55e-21	LNPSITVVTE	PEYNSTSSSFVNRFIEALFYFSAYFDCLE	SCMKDNSNRM
42.	30073.m002204_SCR	683	1.18e-20	RLAPKVVTVV	EQDLSHAGSFLGRFVEAIHYYSALFDSLG	ASYGEESEER
41.	29872.m000537_SCR	309	1.18e-20	LLRPKLITTA	EQDLSHAGSFLGRFVEALHYYSALFDALG	DGLGIDSVER
38.	30131.m007244_SCL3	339	2.05e-20	LSPKLMVITE	QESNHNGFTLMERVTEALNFYAALFDCLE	STVSRPSIER
36.	29790.m000809_SCL3	285	2.05e-20	MSPRLLFLVE	QEADHNLNRLPDRFIEGLHYYSAVFDSID	ATLVGNLGSE

Table S2. *Cont.*

37.	30024.m001746_SCL3	318	7.14e-19	LSPKLMVVME	QDSNHNGSSLMERLLEALYSYAALFDCLE	STVSRTSLER
40.	28492.m000479_SCR	382	1.17e-18	ELSPRIMTLV	EQDISHGGSFLDRFVGSLHYYSTLFDSLG	AFLPCDDSSR
19.	29728.m000844_Os19	322	1.17e-18	LNPSIVILVE	QEGSRSPRSFLSRFMESLHYFAAMYDSLD	DCLPLESSER
39.	29634.m002156_SCL4/7	475	1.43e-18	LNPEIVTLGE	YEASLNQIGYENRFKNALRYYSAVFESLE	PNLSRDSTER
29.	30169.m006225_PAT1	484	7.25e-18	LAPRVVTLVE	QEMNTNTAPFMARVNEGSSYYGALFESIE	STVQRDHTER
5.	30190.m011131_DLT	516	1.83e-17	TSPTIVLMAE	QEAEHNATNLEARVCNSLKYYSAIFDSIN	TSLPLDSLVR
9.	29908.m006159_HAM	355	2.88e-17	SPRLVTLVEE	EVGPVGDGGFVGRFMDSLYHYSALYDSLE	AGFPMQSRAR
4.	30170.m013590_DELLA	320	3.07e-15	IEPEIFTIIE	QESDNNDQGFSYRFNECINYFSFLLESSE	GSTNCLDTYI
6.	29646.m001070_HAM	414	1.04e-12	PEVVVVVDNE	GWGESEPASFRRNFVNGLEFYSMIFESLD	AAAAGGGEWA
30.	29889.m003281_Rc_GRAS	307	1.19e-12	LNPCVMVIIE	PVLNHSSPIFIDSFLEVLLFYSTCFDGVE	GCIDQCDPNR
44.	29661.m000923_SHR	382	4.24e-12	IITIVEEEAD	LDVGIDGLDFVRGFQECLRWFRVYFESLE	ESFSRTSNER
46.	30156.m001710_SHR	310	1.19e-11	LNPCITVIVD	EDSDLDAPDLTSRITTCFNYLWIPFDALE	TFLPTDSCQR
8.	29820.m000986_HAM	381	1.19e-11	GILTFGDGDA	WETVRDCSGFASYFEGNLMHYQALLESIE	MNFPIHLAEA
11.	30174.m008828_HAM	607	1.97e-11	LSPKIVVSLD	RGCDRTDLPFAHHINHSIQSYSGLLESLE	AVNMNIDALQ
43.	29661.m000901_SHR	310	6.37e-11	LDPTIVVLVD	EDADLTSNNLVCRLRSAFNYLWIPYDTVD	TFLPRGSKQR
7.	29820.m000985_HAM	389	1.97e-10	GIVTFGEGIG	HETLSNCDGFGSFFERYMEHYEGLLESIE	WGFPIHLAEA

Motif 4

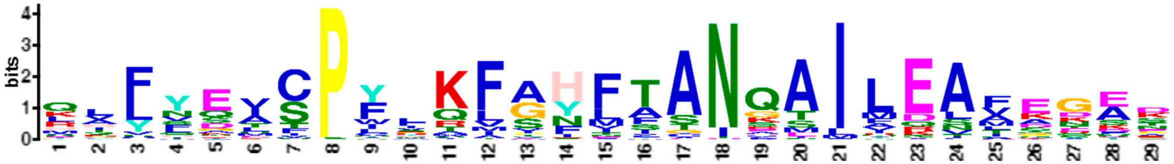

2.	29692.m000540_DELLA	311	7.94e-34	IDHSLSDILQ	MHFYETCPYLKFAHFTANQAILEAFEGKK	RVHVIDFSMN
3.	29807.m000482_DELLA	225	3.83e-31	ASAYENEFLY	HHFYEACPYLKFAHFTANQAILEAFDGHD	CVHVVDFNLM
1.	28677.m000055_DELLA	271	4.98e-30	LDPSYSDTLE	MHFYETCPYLKFAHFTANQAILEAFGTAN	RVHVIDFGLK
23.	30131.m007029_Os43	404	1.81e-26	SNSMEILKIY	QIVYQACPYIKFAHFTANQAIFEAFEAEE	RVHVIDLDIL
39.	29634.m002156_SCL4/7	314	2.64e-25	SSEEDFTLSY	KALNDACPYSKFAHLTANQAILEATEGAS	KIHIVDFGIV
28.	29949.m000124_PAT1	255	1.10e-24	PASADLLSYM	HILYEVCPYFKFGYMSANGAIAEAMKDEN	KVHIIDFQIG
19.	29728.m000844_Os19	159	1.50e-24	PTSEEEFLAF	THLYRVSPYYQFAHFTANQAILEAFEKEE	EEESDSKSRI
25.	27613.m000642_PAT1	246	2.04e-24	PASAELLSYM	HILYEVCPYFKFGYMSANGAIAEAMKDES	RVHIIDFQIA
42.	30073.m002204_SCR	533	9.18e-24	THTQKMASAF	QVFNGISPFVKFSHFTANQAIQEAFERED	RVHIIDLDIM
9.	29908.m006159_HAM	190	3.39e-23	HHQADTLAAF	QLLQDMSPYVKFGHFTANQAILEAVSQDK	RIHIVDYDIM

Table S2. *Cont.*

20.	29929.m004746_Os4	217	6.99e-21	**SFTAEKDEAL**	**RLVYEICPQIQFGYFVANATILEAFEGES**	**SIHVVDLGMT**
41.	29872.m000537_SCR	158	7.92e-21	**TQSQKIFNAF**	**QSYNSISPLIKFSHFTANQAIFQALDGED**	**RVHVIDFDIM**
21.	30147.m013794_Os4	237	1.15e-20	**IASDKKEEAL**	**SLVYEICPHIQFGHFVANSSILEAFEGES**	**FVHVVDLGMT**
27.	29648.m001919_PAT1	266	2.39e-20	**PPSSDRLAAM**	**QILFEICPCFKFGFMAANGAMIESFKGEK**	**GVHIIDFDIN**
38.	30131.m007244_SCL3	126	5.51e-20	**YLVSEEILVQ**	**RLFFELFPFLKLAYVITNQAIVEAMEGEK**	**MVHIIDLNSF**
40.	28492.m000479_SCR	232	8.83e-20	**INHKTVHSAF**	**QVFNNVSPFIKFAHFISNQEILEAFQRRD**	**RVHIIDLDIM**
37.	30024.m001746_SCL3	106	9.92e-20	**TLVSEEILVR**	**KLFFEMFPFLKVGFVITNQAIIEAMEGEK**	**MVHIIDLNAV**
11.	30174.m008828_HAM	451	3.92e-19	**CNLILKIGAY**	**KSFSEISPILQFSNFTCNQALLEACEGSD**	**RIHIVDFDIG**
44.	29661.m000923_SHR	205	7.66e-19	**CSFESTRKMV**	**LKFQEVSPWTTFGHVSCNGAIMEAFEGES**	**KLHIIDISNT**
22.	30169.m006306_Os4	250	1.33e-18	**ITREEKMEAF**	**QLLYQTTPYVAFGFMAANEAICEAARGKD**	**ALHVIDLGMD**
7.	29820.m000985_HAM	207	7.33e-18	**ESFKNFDKAF**	**EVFYQIFPYGMVAHFTANSAILEAKPRDA**	**EILHVVDFDI**
10.	29916.m000544_HAM	505	8.13e-18	**FDVIFKLGAY**	**KVFSEVSPLIQFVNFTCNQALLEALSEAD**	**RIHIIDFDIG**
14.	28650.m000187_LISCL	391	1.11e-17	**TPVSDILKAY**	**QVYVKACPFKRMSNFFANQTIFKLAEKAT**	**RLHIIDFGVL**
15.	28650.m000189_LISCL	366	3.08e-17	**TNTADILKAY**	**QLYVSVCPFRKMSNFFTNRTITKAVEKAT**	**RLHIIDFGIS**
8.	29820.m000986_HAM	204	6.82e-17	**ESSNNFEAAF**	**RAFYQIFPNGRIAHFAANSAILEAMPADA**	**EMIHIVDFDM**
24.	27529.m000047_PAT1	250	1.80e-16	**PVSSDLMTYM**	**GILFKICPYWRFAYTSANVIIREAVEYEP**	**RIHIIDFQIA**
16.	28650.m000190_LISCL	519	5.61e-16	**RTAADILRAY**	**KTHLHACPFKKLSILFANKMIMHAAEKAT**	**TLHIVDFGVS**
29.	30169.m006225_PAT1	318	7.41e-16	**LFAKEHIAST**	**QLLYELSPCFKLGFMAANLAILQSTVDQP**	**NSGTGFHVID**
43.	29661.m000901_SHR	116	1.07e-15	**DAHKFSVIEL**	**ASFVDLTPWHRFGFTAANASILEAIEGFS**	**VVHIVDLSLT**
4.	30170.m013590_DELLA	162	2.65e-15	**FDYSYLNDIQ**	**RYFYKEWSYVKAAHLTANREIFETFAGKK**	**HIHVIDFFIN**
12.	27568.m000253_LISCL	445	5.37e-15	**TTAADVLKAH**	**HLFLAACPFRKLSNFFSNKTIMNIAQNAT**	**TLHIIDFGIL**
5.	30190.m011131_DLT	361	5.37e-15	**RVDDDSGTAW**	**RLLNQVNPIPKFIHFTENEIFLRAFEGKD**	**KVHIIDFDIK**
33.	29889.m003285_Rc_GRAS	288	8.30e-15	**EAIMAPSSTI**	**LASYQEVPFSQVAHFAGIQAIVENVTDAK**	**RIHVIDLGIR**
31.	29889.m003282_Rc_GRAS	220	2.13e-14	**SLVIIPNPSI**	**QACFEGIPFYQAAHLTGIKAIIENVAEAN**	**RSHLLKITAV**
32.	29889.m003284_Rc_GRAS	273	3.51e-14	**PVDPNHNAAS**	**LACHQKIPFIQVARFTAIQEIVENVARAK**	**RIHIIDLEIR**
45.	29706.m001281_SHR	242	3.82e-14	**IDPKFFQRSL**	**LKFYEVSPWFAFPNNIANSSILQVLAQEL**	**DHKRNLHILD**
13.	28650.m000186_LISCL	366	8.00e-14	**TSAAGMLKAY**	**HAYTTACPFQTMSHLYANETIMKLAEKTT**	**RLHIIDFGIL**
18.	29767.m000200_LISCL	446	9.41e-14	**TSAANVLKAY**	**HLYLAACPFRKLTNFLSNKTIMELSANSA**	**SIHIIDFGIL**
17.	28650.m000191_LISCL	469	1.79e-13	**LSAADMLKAY**	**LAYISACPFNKIAIIFANHNILAVSKNAS**	**TLHIIDFGIL**
46.	30156.m001710_SHR	125	2.65e-13	**QRRQMSVTEL**	**AGYVDLIPWHRFGFCASNSAIFKAIEECP**	**KVHILDFSIT**
34.	29910.m000940_Rc_GRAS	295	2.87e-13	**EAMMTPNPTI**	**LASHLETPFCQVAHFAGIQAIVDNVADAK**	**KIHILDLSLR**
6.	29646.m001070_HAM	248	1.06e-12	**SEVVQTIKAY**	**QDFSGISPIPMFNHFPVDQAILETLDDSP**	**PFIHVIDFDI**
35.	30076.m004651_Rc_GRAS	340	4.06e-11	**PDGTSLHLAC**	**LSFHQNVPLNQITQLTSIQAIMENIGSAR**	**KLQVIDLEIR**

Table S2. *Cont.*

Motif 5

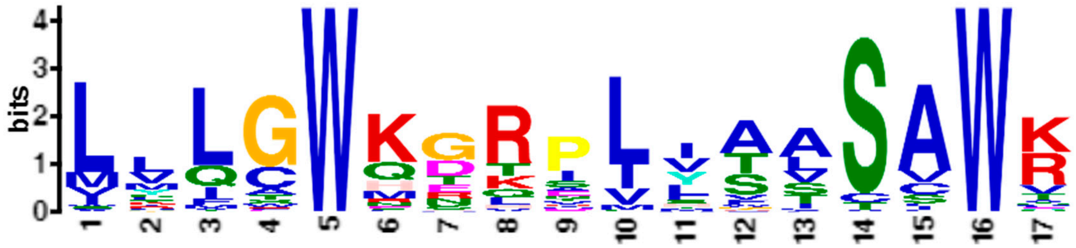

2.	29692.m000540_DELLA	583	5.23e-18	GYRVDENNGC	LMLGWHTRPLIATSAWR	LASKQVVLAQ
18.	29767.m000200_LISCL	722	1.79e-17	NFLINEDGRW	LLQGWKGRIIYALSAWK	PM
34.	29910.m000940_Rc_GRAS	566	3.86e-17	CFTLDRNGKS	LVLGWKGTPLHSLSAWK	FISHEKKGVN
21.	30147.m013794_Os4	509	1.90e-16	GYTVVEEKGC	LVLGWKSKPIVAASCWK	C
1.	28677.m000055_DELLA	546	1.90e-16	GYRVEENNGC	LMLGWHTRPLIATSAWQ	LSDSK
25.	27613.m000642_PAT1	522	2.24e-16	KYTLEERDGA	LYLGWMNRPLIASCAWR	
6.	29646.m001070_HAM	522	3.12e-16	GFYVAKRQAE	LVLCWHERPLIATSVWK	C
22.	30169.m006306_Os4	522	3.66e-16	GYTLASDKGC	LLLGWKGRPIMLASAWK	AHNVPSS
17.	28650.m000191_LISCL	746	6.88e-16	DFVVDQDGQW	MLQGWKGRIIYASSAWV	PA
20.	29929.m004746_Os4	489	9.35e-16	GYTVTEDKGC	LILGWKSKPIIAASCWK	CC
23.	30131.m007029_Os43	669	1.09e-15	GYRLTEDKGC	LLLGWQDRAILAASAWR	C
15.	28650.m000189_LISCL	642	1.96e-15	DFAVDEDGHW	MLMGWKGRIIHAISAWK	PIEE
10.	29916.m000544_HAM	770	1.96e-15	GFHVEKRQAS	LVLCWQRRDLISASAWR	C
16.	28650.m000190_LISCL	797	2.27e-15	DFVIDEDNNW	MLQGWKGRIIYASSCWV	PA
33.	29889.m003285_Rc_GRAS	558	3.01e-15	ACTLSMDGKS	LLIGWKGTPMHSLSAWK	FI
4.	30170.m013590_DELLA	435	3.01e-15	KCTIEENNGC	WMLGWRTRPLIAISAWR	ADNRILISDE
3.	29807.m000482_DELLA	498	3.01e-15	GYCVEENDGC	LTLGWHSRPLIAASAWQ	AVPDIGSPLG
11.	30174.m008828_HAM	716	5.24e-15	GFHVEKRQST	LVLCWQRKELISASAWR	C
42.	30073.m002204_SCR	796	6.87e-15	GYTLVEDNGT	LKLGWKDLCLLTASAWR	PFHATATAAA
41.	29872.m000537_SCR	423	7.86e-15	YTLVEEENGC	LKLGWKDLSLLTASAWK	PSD
37.	30024.m001746_SCL3	432	1.02e-14	GYRIKDENGC	VVICWQDRPLFSLSAWR	CKK
35.	30076.m004651_Rc_GRAS	611	1.33e-14	SCSLDKNGKC	LIVGWKGTPLHSLSAWK	FCGDRLGRFF
26.	28166.m001040_PAT1	443	1.33e-14	HYTLLEKDGA	MLLGWKNRNLVSASAWY	
19.	29728.m000844_Os19	453	1.33e-14	RVFERDEGKT	ISLGWQDRCLLTASAWR	CV

Table S2. *Cont.*

36.	29790.m000809_SCL3	399	1.71e-14	GYKTVSERWS	LMICWHERPIYAVTAWT	C
44.	29661.m000923_SHR	495	2.21e-14	WSMTQSGHAG	IFLCWKEQPVVWASAWK	P
40.	28492.m000479_SCR	498	3.20e-14	GYSLAQGEGA	LRLGWKDTSLFTASAWT	SLASR
13.	28650.m000186_LISCL	643	4.60e-14	NFIVDEDSQW	MLQGWKGRIIYALAVWK	PVHD
38.	30131.m007244_SCL3	453	5.18e-14	GYKIKEENGC	SVICWQDRPLFSVSAWR	FRRYD
14.	28650.m000187_LISCL	668	6.56e-14	DFVVDENGRW	MLQGWKGRVISALSVWK	PVQD
12.	27568.m000253_LISCL	722	1.04e-13	DFVIDEDSRW	LLQGWKGRIVYALSSWE	PDH
27.	29648.m001919_PAT1	543	2.54e-13	RYKLKEEMGA	LLFGWEDKSLIVASAWR	
30.	29889.m003281_Rc_GRAS	421	4.83e-13	SCLLVRNGKC	LMTGWKETPLLSLSAWN	FQQEHSKKNC
29.	30169.m006225_PAT1	599	1.63e-12	GFTVKEDNGG	VCFGWMGKTLTVASAWR	
24.	27529.m000047_PAT1	526	3.85e-12	RYGLQEKDGA	LYLWWMNTAMSSSSAWR	
43.	29661.m000901_SHR	422	6.69e-12	GWGLKKEEDD	LVLTWKGHNVVFATAWM	PT
5.	30190.m011131_DLT	631	7.32e-12	RVKERQDRAA	LTLSWLDQPLYTISAWA	PVDVVGSSSS
9.	29908.m006159_HAM	467	8.01e-12	YRVEELGCNR	LVLSWKSRRLLSASIWT	SPSSDCDL
39.	29634.m002156_SCL4/7	592	2.09e-11	YSLVESQPGF	LSLAWNEVPLLTVSSWR	
46.	30156.m001710_SHR	422	2.70e-11	GWGMKKEEDM	LVLTWKGHNSVFASAWL	PSCLED
8.	29820.m000986_HAM	492	3.76e-11	VRIGGEWNNE	MILEWRGTQLVRVSAWR	N
45.	29706.m001281_SHR	524	7.18e-11	EMRTEEKDGC	VGLWWKGQPVSFCSLWR	LDMKGNDS
32.	29889.m003284_Rc_GRAS	544	9.11e-11	SCTLDVNEKS	LNISWKATPISSLSVWK	FI

Motif 6

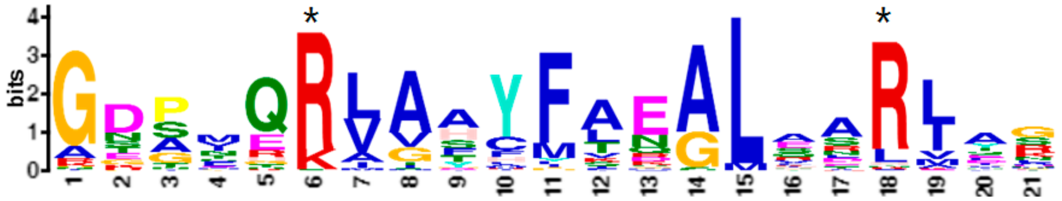

15.	28650.m000189_LISCL	322	8.57e-22	KLIRLHASPY	GDANQRLAHYFANALEARLAG	SGKLMPTLFI
14.	28650.m000187_LISCL	347	8.89e-20	RQIRQHSSPY	GDGNQRLAHYFANALETRLAG	TGTPAYSPLL
17.	28650.m000191_LISCL	425	5.87e-19	KQIRQHSSPF	GDGSQRLAHCFANGLEARLAG	TGAQIYTALS
16.	28650.m000190_LISCL	472	5.87e-19	KQIRQHSSPL	GDGSQRLAHCFANGLEARLAG	SVTGMQSFYT
13.	28650.m000186_LISCL	322	6.91e-19	RQIKQYSSPF	GDGNQRLAHFFANGLEARLAG	TGTPGYAPAV
37.	30024.m001746_SCL3	61	8.13e-19	GQISQLASAE	GDTMQRIAAYFTEALAHRIIK	AWPGVHRALN
23.	30131.m007029_Os43	355	9.54e-19	HHLNRVVTPL	GDSMQRVASCFTEALSARLAA	TLTTQPSNTA
18.	29767.m000200_LISCL	402	2.44e-18	KQIRQHSSPF	GDGNQRLAHCFANGLDARLAG	TGSQIYKGLV

Table S2. *Cont.*

38.	30131.m007244_SCL3	80	3.30e-18	**EHISHLASAD**	**GDTMQRIAAYFTEALADRILK**	**KGWPCLHRAL**
28.	29949.m000124_PAT1	211	1.55e-16	**DELRQIVSVS**	**GEPIQRLGAYMLEGLVARLAS**	**SGSSIYKSLR**
25.	27613.m000642_PAT1	202	1.55e-16	**TELRKMVSVS**	**GEPIQRLGAYMLEGLVARLAS**	**SGSSIYRALR**
12.	27568.m000253_LISCL	401	1.75e-16	**KQIRQNASPT**	**GDGMQRMAHIFADGLEARMAG**	**SGTQIYKAFM**
34.	29910.m000940_Rc_GRAS	245	1.99e-16	**KQCDSISSNT**	**GNPVQRVVYYFAEALHDRIDI**	**ETGKTKSKEL**
39.	29634.m002156_SCL4/7	272	3.25e-16	**IKLRESVCEH**	**GDPTERVAFYFTEALYSRLCL**	**QAEKSLAMFE**
19.	29728.m000844_Os19	116	5.92e-16	**SELYTSVCLT**	**GDSVQRVVAYFADGLSARLLT**	**RKSPFYEMIM**
2.	29692.m000540_DELLA	274	6.66e-16	**KQIGFLAVSQ**	**AGAMRKVATYFAEALARRIYR**	**LYPQSPIDHS**
30.	29889.m003281_Rc_GRAS	98	8.43e-16	**NLCDFLSSNK**	**GNSVQRVVHYFTKALQDRISQ**	**ESETVSSKRS**
1.	28677.m000055_DELLA	234	1.19e-15	**KHIGLLAASQ**	**ASSMRKVATYFAEALARRIYK**	**IYPQESLDPS**
42.	30073.m002204_SCR	488	1.68e-15	**LEISQLSTPY**	**GTSAQRVAAYFSEAMSARLIN**	**SCLGIYATLP**
35.	30076.m004651_Rc_GRAS	293	2.36e-15	**SRCEWIASQR**	**ANPVQRVVYCFAEALRERIDK**	**ATGRFIPKER**
24.	27529.m000047_PAT1	206	6.31e-15	**HVLEQMVSVS**	**GEPIQRLGAYMLEGLRARVEL**	**SGSKIYRALK**
27.	29648.m001919_PAT1	222	1.07e-14	**NELRQMVSIQ**	**GDPSSRIAAYMVEGLAARMAA**	**SGKYLYKALK**
33.	29889.m003285_Rc_GRAS	242	1.32e-14	**NQCDRLSSST**	**GNAVERVVHYFCKALRERIDR**	**ETGKSLGKQH**
32.	29889.m003284_Rc_GRAS	227	1.32e-14	**NHCELLSSNI**	**GNPVQRVVYHFSEALKERIDR**	**ETGRFPSIEY**
31.	29889.m003282_Rc_GRAS	181	2.00e-14	**EFCDQSSSNA**	**GNPVQRVVYYFFGALRRRIDQ**	**ELGKITSRSL**
36.	29790.m000809_SCL3	69	2.45e-14	**SEISELSSVS**	**GDSMQRLAARFASALAIRLVK**	**RWPGLYKALN**
20.	29929.m004746_Os4	169	3.32e-14	**SELRANALVF**	**GTSFQRVASCFVQGLSDRLTL**	**LQPLGAVGVL**
6.	29646.m001070_HAM	206	5.45e-14	**RLNHRLQSPV**	**GKPLQRAAFFFKEALQNLLAG**	**SPRTPTHPTS**
41.	29872.m000537_SCR	111	1.08e-13	**PEISELSSPF**	**GSSFERVGSYFAHALQARVVS**	**SCLGTYSPLT**
21.	30147.m013794_Os4	188	1.57e-13	**SELRSSALVF**	**GSSFQRVASCFFQGLADRLSL**	**VQPLGTVSLV**
9.	29908.m006159_HAM	139	1.73e-13	**RLKELVSPND**	**GTNMERLAAYFTDALQGLLEG**	**HGTSTKHLIH**
26.	28166.m001040_PAT1	233	2.29e-13	**EKARAAVSIS**	**GEPIQRLGAYLVEGLVARKES**	**SGNNIYHALR**
22.	30169.m006306_Os4	200	8.94e-13	**AQIWASVNPF**	**GDSLQRVSYCFALGLRSRLSL**	**LQNATSNGTF**
5.	30190.m011131_DLT	315	9.77e-13	**AKLGELSSPK**	**GTAVSRLIAYYTEALALRVTR**	**LWPHIFHIST**
43.	29661.m000901_SHR	66	1.16e-12	**WVLNNIAPPD**	**GDSNQRLTCAFLRALIARAAK**	**SGTCKLLAAM**
46.	30156.m001710_SHR	80	1.27e-12	**WVLNNVASLV**	**GDPNQRLTSWFLRALISRASK**	**VCPTAMNFDG**
10.	29916.m000544_HAM	459	1.51e-12	**ARLNQQLSPI**	**GKPLHRAAFYFKEALQLLLLM**	**NNNPVTSLPP**
11.	30174.m008828_HAM	409	2.32e-12	**RLNHQLSLSI**	**GKPHTRAAFYFKEALQLLLHM**	**NNTANPSSLS**
45.	29706.m001281_SHR	197	4.96e-12	**YVLHELASST**	**GDANHRLAFYGLQALTHHLSS**	**SRTSASIGSV**
44.	29661.m000923_SHR	158	1.54e-11	**WMLNELGSPY**	**GDIDKKLASYFLQALFSRMTD**	**SGERCYRTLA**
40.	28492.m000479_SCR	192	2.68e-11	**ELTQMASPYG**	**PSCAERVVAYFAKAMASRVIN**	**SWLGICSPLI**
29.	30169.m006225_PAT1	278	4.26e-11	**TRVSQVSNPR**	**GNSEQRLMEYMSMALKSRLNS**	**ADNPPPVAEL**
4.	30170.m013590_DELLA	126	6.72e-11	**ILSQILVNSK**	**ARATQSMAALFAEAMSSRVYR**	**LYPQYFDYSY**
3.	29807.m000482_DELLA	184	1.16e-9	**MQALLTRVNT**	**NSGIGKVAGCFIDALSLRIFS**	**PVNGVGVAVG**

Table S2. *Cont.*

Motif 7

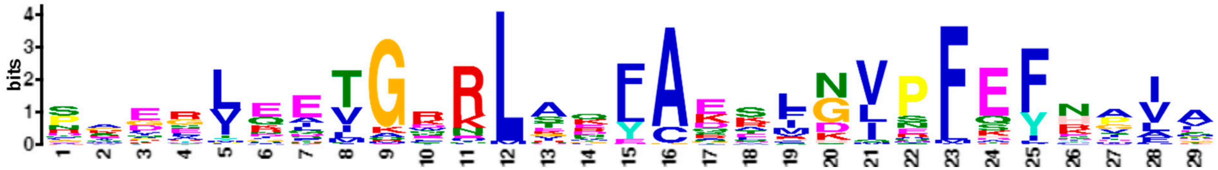

18.	29767.m000200_LISCL	519	5.05e-29	GIDFPQPGFR	PAERVEETGRRLATYAKKFNVPFEYNAIA	KKWETIKFEE
12.	27568.m000253_LISCL	519	4.48e-28	GIDFPHPGFR	PAERVEETGHRLSNYAKKFNVPFEFNAIA	QKWDTVQIEQ
16.	28650.m000190_LISCL	593	8.41e-27	GIELPQQGFR	PAERIEETGRRLARYCERFNVPFEYNSIA	AQNWENIRIE
15.	28650.m000189_LISCL	440	1.33e-26	GIDYPQPGFR	PGERVEETGRRLKRLADKLNVPFEYNAIA	QKWETIQGED
17.	28650.m000191_LISCL	543	4.97e-26	GIELPQSGFR	PGERVQETGRRLAKYCELHKVPFEYNAIA	KKWETIQIDD
14.	28650.m000187_LISCL	465	2.62e-25	GIELPQPGFR	PAERVEETGRRLQRYCERFNVPFKYHAVA	QKWETIKYED
13.	28650.m000186_LISCL	440	2.20e-24	GIEFPQPGFR	PAERVEETGRRLSKYCERFNVPFEYDSIA	QNWESIQYED
5.	30190.m011131_DLT	429	1.03e-21	SHVRITGIGE	SKQELNETGDRLAGFAEALNLPFEFHPVV	DRLEDVRLWM
27.	29648.m001919_PAT1	340	1.02e-20	GIDDPESVQR	STGGLKIIGQRLEKLAEALKVPFEFHAVA	SKTSLVSPSM
23.	30131.m007029_Os43	472	1.54e-20	PFLRITGVGS	CIESVRETGRCLTELAHSLHVPFEFHPVA	EELEDLKPHM
42.	30073.m002204_SCR	601	2.30e-20	PYVRLTGLGT	SIEALEATGKRLSDFAQKLGLPFEFFPVA	DKVGNLDPDR
32.	29889.m003284_Rc_GRAS	343	3.43e-20	LLKISAIGTT	SKHLIEDTGKRLASFAESMNVPFSFRAVM	VSDMLDLKKE
41.	29872.m000537_SCR	226	6.60e-20	RSMRITGFGS	SSELLESTGRRLADFASSLGLPFEFHPLE	GKIGSVSDIS
28.	29949.m000124_PAT1	329	4.85e-19	GIDDSTSAYA	RGGGPNIVGKRLAKLAESVKVPFEFHAAA	MPNSEVHIKN
24.	27529.m000047_PAT1	324	4.85e-19	GVDDPQSAYA	RGGGLDIVGKRLSSFAESHNVPFQFHDAA	MSGCEVQLEH
2.	29692.m000540_DELLA	383	6.17e-19	LTGIGPPSHD	NSDHLQEVGWKLAQLAETIHVEFEYRGFV	ANSLADLDAS
25.	27613.m000642_PAT1	320	1.25e-18	GIDDSTSAYA	RGGGLDIVGQRLSRLAESCKVPFEFHAAG	VSGSEIELKN
40.	28492.m000479_SCR	300	1.58e-18	PHIRMTGMGT	SMDLLVETGKQLSNFAKRLGLSFEFHPIA	KKFGEIDVSM
1.	28677.m000055_DELLA	343	2.23e-18	LTGIGPPQSN	NTDALQQVGWKLAQLADTIGVEFEFRGFV	ANSLADLQPE
38.	30131.m007244_SCL3	194	1.05e-16	PHLRITGIHE	QKEVLEQMALRLTEEAEKLDIPFQFNPIM	SKLEDLDIES
4.	30170.m013590_DELLA	234	1.56e-16	ISGIGFPNHD	NSDYLKSVGWKLAQLAETLNIDFEYRGFL	AYNLADLDAA
33.	29889.m003285_Rc_GRAS	358	1.90e-16	LLKITAVGTT	SKHLIKDTGKRLTSFAESISLPFAFNIVM	VSDMLDLTED
35.	30076.m004651_Rc_GRAS	411	2.31e-16	LKVTALGLTG	KGKKIEETGRSLESFAKSMNIPFTFKAVY	VSCMKNIKEK
37.	30024.m001746_SCL3	174	4.53e-16	PHLRITGIHQ	QKEVLDQMAHKLSEEAERLDIPFQFNPIV	SKLENLDIEK
9.	29908.m006159_HAM	267	4.53e-16	LSRGGNSGKR	SIGTVQETGRRLVAFAASIGQPFSFHHCR	LDSDESFRPS
39.	29634.m002156_SCL4/7	389	7.26e-16	GIPAPVLGKS	PAASLLATGNRLGDFAKLLDLNFEFVPIL	TPIQELNESN
46.	30156.m001710_SHR	200	2.00e-15	PFTRPQVPPW	LNVSTEEVGLRLGNFAKSRDVPFEFIVID	DPSSDILCKE

Table S2. *Cont.*

34.	29910.m000940_Rc_GRAS	365	2.19e-15	**HLKITAIGTT**	**SRELIENTGKRLISFAETMNIAFSFKMAL**	**VSDLLDLKED**
44.	29661.m000923_SHR	291	3.13e-15	**GGGGNCGLVS**	**AQKVMKEIGSRMEKFARLMGVPFKFNVIH**	**HAGDLCDLNL**
3.	29807.m000482_DELLA	297	8.94e-15	**LTGIGPPSPD**	**GRDSLREIGVKLAELARSVNVRFAFRGVA**	**ASRLEDVKPW**
20.	29929.m004746_Os4	290	1.37e-14	**RCLRITGVGN**	**SAERLQALGDELDCYARSLGLNFEFLWVE**	**SSLEKLKSTD**
30.	29889.m003281_Rc_GRAS	221	4.38e-14	**LKISGVGVKA**	**SRQRIEDAGKSLACFANTLDLPFSFKTII**	**VASIKDLKKD**
29.	30169.m006225_PAT1	395	2.13e-13	**ITAVADNSAE**	**EKERLKVVGTTLSQLAEQFGVSLHFNVVS**	**AKLGDLSRES**
43.	29661.m000901_SHR	191	1.83e-12	**AGCTEDIPPM**	**LDLSYEELGCKLINFARSRNVIMEFRVVD**	**SSYADGFSSL**
21.	30147.m013794_Os4	310	2.12e-12	**CRLRITAVGL**	**CVGRFQTIGDELVEYAKDVGINLEFSVVE**	**STLENLQPDD**
7.	29820.m000985_HAM	293	3.72e-12	**ITAIKWREEH**	**PWRKLEETKRRLQNYADSLGLMLHVEEIE**	**VQNLAREIKR**
36.	29790.m000809_SCL3	182	4.91e-12	**PHLKFTCLNT**	**DKAILDKLGQRLVKEAEASDMAFQFHPLN**	**ISLRDLTADM**
22.	30169.m006306_Os4	321	3.63e-11	**RITGLINDHQ**	**NLLELEASMKVLAEDASSLGVSLEFNMIL**	**ESVTPSLLTR**
11.	30174.m008828_HAM	522	9.99e-11	**KITAFVSPSH**	**DEIELGFTQENLRVFAGEINMPFELEILG**	**LDALNSGSWS**
6.	29646.m001070_HAM	325	9.99e-11	**LRITAVVTED**	**YAIQTQLIKQCLSQYALELKIRFQIEFVL**	**TRTFEMVSFK**
10.	29916.m000544_HAM	576	2.21e-10	**ITAFASPSTH**	**HPVEVLLMRENLTQFANEIGISFELDVIN**	**FDSLEQSCYS**
Motif 8						
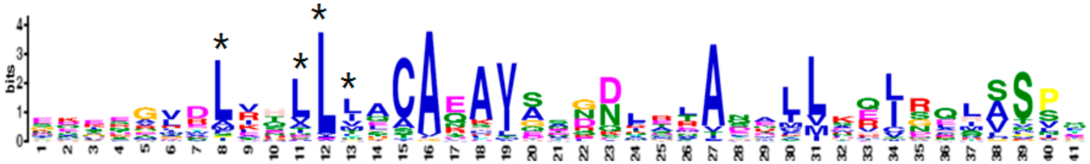						
17.	28650.m000191_LISCL	384	3.82e-34	**GGKARAKRQG**	**NKKEVVDLRTLLILCAQAVSSDDRRTANEILKQIRQHSSPF**	**GDGSQRLAHC**
18.	29767.m000200_LISCL	361	2.09e-31	**GGKGRGKKHN**	**GKKEVVDLRTLLISCAQAVAADDRRSAYEMLKQIRQHSSPF**	**GDGNQRLAHC**
16.	28650.m000190_LISCL	431	1.12e-30	**GGKTRSKKQS**	**KKKETVDLRSLLILCAQAVSGNDFRTANELVKQIRQHSSPL**	**GDGSQRLAHC**
2.	29692.m000540_DELLA	233	9.91e-28	**PTTRPVVLVD**	**SQENGIRLVHLLMACAEAVQQNNLTLAEALVKQIGFLAVSQ**	**AGAMRKVATY**
12.	27568.m000253_LISCL	360	1.40e-27	**SGKGRGKKQK**	**GKSNVVDLRTLLTLCAQAVAADDRRNTNDLLKQIRQNASPT**	**GDGMQRMAHI**
42.	30073.m002204_SCR	447	1.04e-26	**EKKEEQRQQK**	**RDEEGLHLLTLLLQCAEAVSADNFEEANKMLLEISQLSTPY**	**GTSAQRVAAY**
1.	28677.m000055_DELLA	193	8.27e-26	**EPNRPMVLID**	**SQETGVRLVHTLLACAEAIQQDNFKLAEALLKHIGLLAASQ**	**ASSMRKVATY**
28.	29949.m000124_PAT1	170	1.31e-25	**PEMDIWRQMM**	**EAISRGDLKHVLIACAKAVSDNDLLMAQWLMDELRQIVSVS**	**GEPIQRLGAY**
15.	28650.m000189_LISCL	281	3.87e-24	**PNNTRMRRHA**	**KDKKMFDLSTLLIQCAQATGTGDQRTAYQQLKLIRLHASPY**	**GDANQRLAHY**
14.	28650.m000187_LISCL	306	4.46e-24	**GRTARGRRQG**	**NKGEVVDLSTLLAQCAQSVSISDHRTATELLRQIRQHSSPY**	**GDGNQRLAHY**
13.	28650.m000186_LISCL	281	2.34e-23	**RTARTKKRGS**	**NKRDMMDLWTVLPQCAQAVANDDQTTAKELLRQIKQYSSPF**	**GDGNQRLAHF**
22.	30169.m006306_Os4	159	1.69e-22	**MVPSMEEVSY**	**GVDRGLHLVHLLLACAEAVGCRDTQLANSVLAQIWASVNPF**	**GDSLQRVSYC**
20.	29929.m004746_Os4	128	1.69e-22	**MISKKNEIKE**	**EDIDGMKLVQQLIACAEAVACRDKTHASALLSELRANALVF**	**GTSFQRVASC**

Table S2. *Cont.*

41.	29872.m000537_SCR	70	2.83e-22	**AIKEGEEEEE**	**EDSTGLRLLGLLLQCAECVAMDNLDEAADLLPEISELSSPF**	**GSSFERVGSY**
21.	30147.m013794_Os4	147	2.83e-22	**SEEGENHGEG**	**GNTDGMRLVQLLIACAEAVACRDKSHASALLSELRSSALVF**	**GSSFQRVASC**
23.	30131.m007029_Os43	314	2.33e-21	**MVPLPIGPEQ**	**EQDSGLQLVHLLLACAEAVAKEDYMLARKYLHHLNRVVTPL**	**GDSMQRVASC**
24.	27529.m000047_PAT1	165	8.67e-21	**QARWDWTRME**	**EMIPRLDTRQILFACAQAISDGDISRAAALMHVLEQMVSVS**	**GEPIQRLGAY**
25.	27613.m000642_PAT1	161	9.75e-21	**SEEEKWKFLM**	**ETVSRRDLKEVLYACAQAIDSNDMLTVEWLMTELRKMVSVS**	**GEPIQRLGAY**
36.	29790.m000809_SCL3	28	1.55e-20	**LSSASPSPYE**	**TQERGIRLIQLLLKCANDASSGNLHRADACLSEISELSSVS**	**GDSMQRLAAR**
40.	28492.m000479_SCR	150	2.46e-20	**GASNGRGLSR**	**IDEHGLNLISLLFECAVAISVDNLGEAHRMLLELTQMASPY**	**GPSCAERVVA**
38.	30131.m007244_SCL3	39	3.88e-20	**SPYPWLRELK**	**SEERGLYLIHLLHACASHVAAGSVENANICLEHISHLASAD**	**GDTMQRIAAY**
43.	29661.m000901_SHR	25	3.53e-19	**GFPTSKALGN**	**NFGDANCMEQLLVHCANAIESNDATLAQQILWVLNNIAPPD**	**GDSNQRLTCA**
27.	29648.m001919_PAT1	181	3.94e-19	**SSSTSSNKEV**	**SQLSPRAPKRLLLECANALAEDNIEAADALINELRQMVSIQ**	**GDPSSRIAAY**
35.	30076.m004651_Rc_GRAS	252	5.42e-19	**FGYALSGLSE**	**EETRDVELAHLLLAAAEKVGYQQFDRASRLLSRCEWIASQR**	**ANPVQRVVYC**
37.	30024.m001746_SCL3	20	1.02e-18	**SPYPWLRELK**	**SEERGLYLIHLLLTCGNHVAAGSLENAEIALGQISQLASAE**	**GDTMQRIAAY**
45.	29706.m001281_SHR	156	2.88e-18	**KGAANNANSG**	**NNKEGRWAEDLLNPCAAAVSTGNLSRVQHLLYVLHELASST**	**GDANHRLAFY**
32.	29889.m003284_Rc_GRAS	186	4.80e-18	**FGLFFSDLSD**	**DESRNVELVELLLASAEKVGYQQFERASRLLNHCELLSSNI**	**GNPVQRVVYH**
5.	30190.m011131_DLT	274	9.68e-18	**TEEAAGANHQ**	**EEYQAYELVSLLTACVEAIGSKNMAVINHCIAKLGELSSPK**	**GTAVSRLIAY**
33.	29889.m003285_Rc_GRAS	201	3.14e-17	**FDLSFSGLSV**	**DEAKKVELAELLLLSAEKVGNQQYERASILLNQCDRLSSST**	**GNAVERVVHY**
19.	29728.m000844_Os19	75	2.74e-16	**KPDQKKKGVV**	**EDGKGLHLIHLLLITATAVDENNVATALENLSELYTSVCLT**	**GDSVQRVVAY**
46.	30156.m001710_SHR	39	6.22e-16	**SLSGALRGCL**	**GSLDGACIEKLLLHCASALESNDGTLAQQVMWVLNNVASLV**	**GDPNQRLTSW**
10.	29916.m000544_HAM	418	1.16e-15	**EVGGGHQQQQ**	**QAQHQHALLDQLYKAAELVGTGNFSHAQGILARLNQQLSPI**	**GKPLHRAAFY**
30.	29889.m003281_Rc_GRAS	57	1.39e-15	**FDGSQFGLSG**	**EEIKDVELSLLLLASAHRVGNRQFDHASKLLNLCDFLSSNK**	**GNSVQRVVHY**
3.	29807.m000482_DELLA	141	1.65e-15	**QNQLRVVTAT**	**EEDSAIRLVHLLMTCAESIQRGDLSLAGSLVEDMQALLTRV**	**NTNSGIGKVA**
34.	29910.m000940_Rc_GRAS	204	1.81e-15	**FDLYFSGLSD**	**EDAKHVELAESLLASAERIGNQQYDSASRLLKQCDSISSNT**	**GNPVQRVVYY**
44.	29661.m000923_SHR	117	7.80e-15	**FSFSPSHDLN**	**FDFSSRWATDILLETARAIADKNSARVQQLMWMLNELGSPY**	**GDIDKKLASY**
26.	28166.m001040_PAT1	192	1.19e-14	**YHKGIEETGL**	**QSIPPDNLKQLLIACAKALSENNINEFDRLIEKARAAVSIS**	**GEPIQRLGAY**
31.	29889.m003282_Rc_GRAS	140	1.16e-13	**AGQGLSTEEI**	**KRQNMWSLLNFFLASAEKVSNGKFDRASRLLEFCDQSSSNA**	**GNPVQRVVYY**
7.	29820.m000985_HAM	132	1.85e-13	**QSLIMPKECM**	**GISNLFSLTNLAMAYAEATENKQAALAEVIMRRIGEKVSPI**	**SGIKERLLYY**
29.	30169.m006225_PAT1	237	3.43e-13	**PTSSSSSTSV**	**TTPLPNYSKHTLVEAASAIYDGKTEVASEILTRVSQVSNPR**	**GNSEQRLMEY**
6.	29646.m001070_HAM	164	3.44e-12	**SLDLSHNIGN**	**WSNFGFDLIQELIRAADCIDSSEIQLANVILDRLNHRLQSP**	**VGKPLQRAAF**
11.	30174.m008828_HAM	367	9.98e-12	**KQKIMTDELA**	**AQQLQQAIINPICQAAELIETGNPALAQGILARLNHQLSLS**	**IGKPHTRAAF**
4.	30170.m013590_DELLA	264	1.75e-8	**IDFEYRGFLA**	**YNLADLDAAMLELRTHEAIAVNAVFALHKLLARPGDIHKLL**	**SMVKHIEPEI**

Table S2. *Cont.*

Motif 9

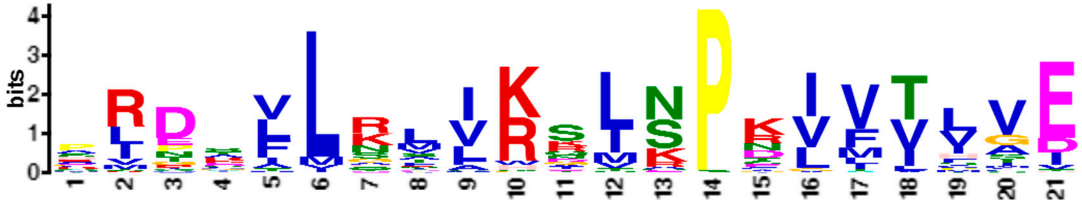

26.	28166.m001040_PAT1	309	4.58e-21	HSDESVDVSN	PRDGLLRMIKSLNPKVVTLVE	QESNTNTTPF
28.	29949.m000124_PAT1	397	1.94e-20	LPDESVSTQN	HRDRLLRLVKSLSPKVVTLVE	QESNTNTAAF
25.	27613.m000642_PAT1	388	1.94e-20	MPDESVGTQN	HRDRLLRLVKSLSPKVVTLVE	QESNTNTAPF
24.	27529.m000047_PAT1	392	2.05e-18	MPDESVSTWN	HRDRLLRLVKSLSPKVVTLIE	QESNTNTKPF
27.	29648.m001919_PAT1	408	4.94e-18	MPDESVSTVN	ERDQLLRMVKSLNPKLVTVVE	QDVNTNTAPF
3.	29807.m000482_DELLA	366	3.50e-17	LLGSDPNRNL	SIDMVLNWIRNLNPKIMTVVE	QEANHNQPGF
2.	29692.m000540_DELLA	450	8.14e-16	ELHKLLARPG	AIDKVLSVVKQMKPEIVTIVE	QEANHNGPVF
29.	30169.m006225_PAT1	463	2.58e-15	MPDESVSTEN	PRDELLRRVKGLAPRVVTLVE	QEMNTNTAPF
1.	28677.m000055_DELLA	410	8.60e-15	ELHRLLARPG	GMEKVLSSIKAMKPKIVTVVE	QEASHNGPVF
17.	28650.m000191_LISCL	611	1.91e-14	LLDETVVVNS	PRNAVLNLIRKMSPDIFIHAI	VNGSYSAPFF
14.	28650.m000187_LISCL	533	2.98e-14	LPDDTVVANS	ARDAVLKLIRKIRPDIFIHGV	INGTYNAPFF
15.	28650.m000189_LISCL	508	3.70e-14	LPDDTIVLDS	PRDAVLRLIKSINPVIFLHGV	VNGSYNAPFF
18.	29767.m000200_LISCL	587	4.58e-14	LLDETVVVDS	PKDIVLRLVKKINPNIFILGI	INGAYSAPFF
39.	29634.m002156_SCL4/7	454	5.10e-14	LYNLLDETYG	AVETALKMAKSLNPEIVTLGE	YEASLNQIGY
46.	30156.m001710_SHR	289	2.13e-13	EQKCRAQDSS	LRDTFLHSIKILNPCITVIVD	EDSDLDAPDL
37.	30024.m001746_SCL3	297	2.13e-13	SSPLSSTASV	KMDYFLNTLWSLSPKLMVVME	QDSNHNGSSL
19.	29728.m000844_Os19	301	2.85e-13	SHLNTLNDFL	KIPDTLKSIHSLNPSIVILVE	QEGSRSPRSF
13.	28650.m000186_LISCL	508	5.05e-13	IPDDTMVVNS	MRDSILKLMRRINPDIFIHGV	VNGTYNAPFF
34.	29910.m000940_Rc_GRAS	430	6.67e-13	LPRNLISLPN	RLDSMMRMIKNVNPCVIVVAE	VEANHNSPTF
16.	28650.m000190_LISCL	662	6.67e-13	LLDEIVEVDC	PRNAVLDLIRKIKPNIYVHCI	INGSYNAPFF
10.	29916.m000544_HAM	641	1.05e-12	PVWSASNQPA	ALPSLLRFVKQLSPKIVVSLD	RGDRTDLPFP
12.	27568.m000253_LISCL	587	1.26e-12	LLDETVVVES	PRTNVLNLIREMNPDVFITGI	VNGAYNAPFF
43.	29661.m000901_SHR	289	2.33e-12	ESSSSSSMSS	LRTMFLKSLRSLDPTIVVLVD	EDADLTSNNL
11.	30174.m008828_HAM	586	2.33e-12	PIAPFSNYPS	SLPVVLRFVKQLSPKIVVSLD	RGCDRTDLPF
23.	30131.m007029_Os43	534	2.54e-12	VNRLHHVPGN	CLPNLLAMIRDQAPNIVTIVE	KEASHNGPYF
32.	29889.m003284_Rc_GRAS	408	4.22e-12	FLMNLLVAPN	RLESIMGMLRNINPNVMVVME	VEANNNSPSF

Table S2. *Cont.*

35.	30076.m004651_Rc_GRAS	476	6.37e-12	FLRTLISSPE	CLENLMRVIKNLKPSIMVINE	IEANHNSPNF
22.	30169.m006306_Os4	388	6.92e-12	LHKFVKESRG	SLKAILQAIKRLSPTLLTVVE	QDANHNGPFF
30.	29889.m003281_Rc_GRAS	286	7.50e-12	ILRFIKAQQD	CLQCLLTVLRKLNPCVMVIIE	PVLNHSSPIF
33.	29889.m003285_Rc_GRAS	423	8.13e-12	LLRSLISLPD	RLNSVMKVIRILNPSITVVTE	PEYNSTSSSF
45.	29706.m001281_SHR	394	1.21e-11	LHHLNHNNPD	ERTEFLKVLRSLEPKGVILSE	NNTDRCQICG
21.	30147.m013794_Os4	375	1.31e-11	LHCVVKESRG	ALNSVLQTIHALSPKILALVE	QDSSHNGPFF
38.	30131.m007244_SCL3	318	1.66e-11	LSPLSLAPSP	KMGSFLNALWGLSPKLMVITE	QESNHNGFTL
20.	29929.m004746_Os4	355	2.84e-11	LHCAVKESRG	ALNTVLQILHELSPKLLILVE	QDSGHNGPFF
5.	30190.m011131_DLT	495	2.84e-11	HKTLYDGNGG	ALRDFLGLIRSTSPTIVLMAE	QEAEHNATNL
44.	29661.m000923_SHR	357	3.83e-11	SLHSVTTVAN	RRDYIISNFRRLQPRIITIVE	EEADLDVGID
36.	29790.m000809_SCL3	264	9.88e-11	NRSTCIKDCK	QMSDFLAMVRSMSPRLLFLVE	QEADHNLNRL
9.	29908.m006159_HAM	333	1.22e-10	LPHFSYRAPD	SVASFLSGSKTLSPRLVTLVE	EEVGPVGDGG
6.	29646.m001070_HAM	391	1.61e-10	TFRRLGSSNN	NINSFVTDIRRVSPEVVVVVD	NEGWGESEPA
				Motif 10		
28.	29949.m000124_PAT1	365	1.05e-25	AAAMPNSEVH	IKNLGVEPGEALAVNFAFMLHHLPDESVS	TQNHRDRLLR
24.	27529.m000047_PAT1	360	2.34e-25	DAAMSGCEVQ	LEHLCVRPGEAVVVNFPYVLHHMPDESVS	TWNHRDRLLR
27.	29648.m001919_PAT1	376	4.38e-25	AVASKTSLVS	PSMLDCKAGEALVVNFAFQLHHMPDESVS	TVNERDQLLR
25.	27613.m000642_PAT1	356	5.96e-25	AAGVSGSEIE	LKNLGIRPGEALAINFALMLHHMPDESVG	TQNHRDRLLR
12.	27568.m000253_LISCL	555	1.71e-24	AIAQKWDTVQ	IEQLKIDKNEVLVVNCLYRLRNLLDETVV	VESPRTNVLN
14.	28650.m000187_LISCL	501	1.28e-23	AVAQKWETIK	YEDLNIDRGEMTVVNCLYRLRNLPDDTVV	ANSARDAVLK
17.	28650.m000191_LISCL	579	1.33e-21	AIAKKWETIQ	IDDLKLNHGEVVAVNCLFRSKNLLDETVV	VNSPRNAVLN
13.	28650.m000186_LISCL	476	2.71e-21	SIAQNWESIQ	YEDFKIDRNEMIVVNCLYRLKNIPDDTMV	VNSMRDSILK
39.	29634.m002156_SCL4/7	425	6.85e-21	PILTPIQELN	ESNFRVDPDEVLAVNFMLQLYNLLDETYG	AVETALKMAK
15.	28650.m000189_LISCL	476	6.93e-20	AIAQKWETIQ	GEDLQIDKDEVVAVCCMNRLKNLPDDTIV	LDSPRDAVLR
16.	28650.m000190_LISCL	630	1.30e-19	IAAQNWENIR	IEELKINSNEVLAVNCLARFKNLLDEIVE	VDCPRNAVLD
29.	30169.m006225_PAT1	431	1.97e-19	VVSAKLGDLS	RESLGCEPEEPLAVNFAFNLYRMPDESVS	TENPRDELLR
5.	30190.m011131_DLT	465	1.95e-18	PVVDRLEDVR	LWMLHVKEGESVAVNCVFQMHKTLYDGNG	GALRDFLGLI

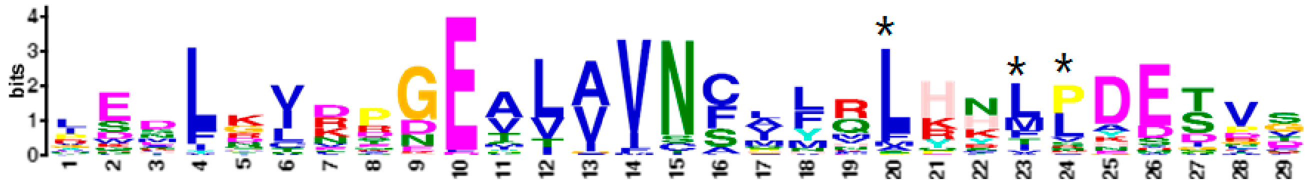

Table S2. *Cont.*

21.	30147.m013794_Os4	346	4.18e-18	VVESTLENLQ	PDDIKVFDGEVLVVNSILQLHCVVKESRG	ALNSVLQTIH
3.	29807.m000482_DELLA	334	1.16e-17	VAASRLEDVK	PWMLQVNPKEAIAVNSIMQLHKLLGSDPN	RNLSIDMVLN
18.	29767.m000200_LISCL	555	1.82e-17	AIAKKWETIK	FEELKIDREEIIVVTCFYRAKNLLDETVV	VDSPKDIVLR
22.	30169.m006306_Os4	359	8.17e-17	LESVTPSLLT	RENLNLRDGEALFFNSIMHLHKFVKESRG	SLKAILQAIK
38.	30131.m007244_SCL3	230	3.73e-16	PIMSKLEDLD	IESLRVKTGEALAVSSVLQLHSLLAADDE	LLKQKSPATK
37.	30024.m001746_SCL3	210	1.47e-15	PIVSKLENLD	IEKLRVKTGEALAISSVLQLHSFLASDDE	LRKRSPVTLK
46.	30156.m001710_SHR	254	3.49e-15	HFESLLNHLS	PSALNLRDDEALVINCQNWLRYLSNEQKC	RAQDSSLRDT
9.	29908.m006159_HAM	304	5.53e-15	CRLDSDESFR	PSALKLVRGEALIMNCMLHLPHFSYRAPD	SVASFLSGSK
20.	29929.m004746_Os4	326	1.83e-14	WVESSLEKLK	STDFKLLDGEVVIINSILQLHCAVKESRG	ALNTVLQILH
43.	29661.m000901_SHR	236	1.27e-13	FSSLIEQLRV	QNLVYTDSGEALVINCHMLPHYIPEETLS	GIHNTTNSSN
36.	29790.m000809_SCL3	218	2.06e-13	PLNISLRDLT	ADMLQVASGEALAFISILNLHLLLAEDDR	VDAHFGGNRS
45.	29706.m001281_SHR	365	3.07e-12	LDNHPLQSLN	AQVINTNPEDTLVVCAQFRLHHLNHNNPD	ERTEFLKVLR

Motif 11

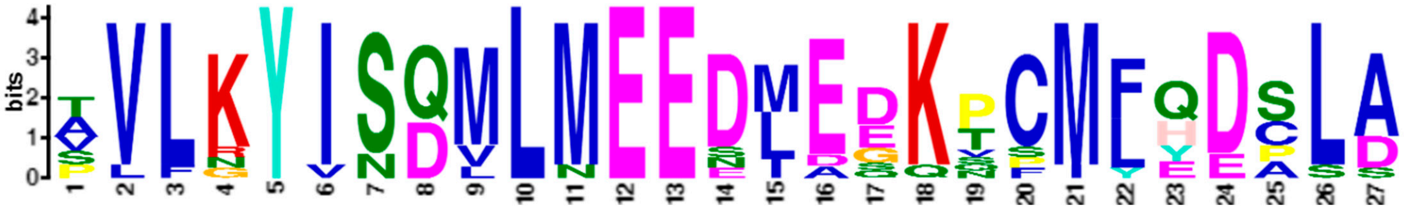

15.	28650.m000189_LISCL	69	4.51e-33	YNSETSNFSQ	AVLKYISDMLMEEDLEDKPCMLQDCLA	LQAAEKSLYD
14.	28650.m000187_LISCL	77	1.29e-31	SSEGHDPNNN	AVLKYISDMLMEEDLEGKTCMLQDCLA	LQAAEKSLYD
17.	28650.m000191_LISCL	84	1.86e-30	SPSDDNDFSE	TVLNYISQMLMEEDMEQKPCMFHDPLA	LQAAERSLYD
18.	29767.m000200_LISCL	82	1.02e-29	DSPEDCDFSD	TVLRYISQMLMEEDIEDKTCMLQDSLD	LQAAEKSFYE
12.	27568.m000253_LISCL	80	2.35e-28	DLHEDFDFSD	VVLKYISDMLMEEDIEEKTCMFQESSA	ALQAAEKSLY
16.	28650.m000190_LISCL	111	2.01e-27	SPSDDSDSSD	PVLKYISQMLMEENMEDKPPMYYDPLD	LKATEKSLYD
13.	28650.m000186_LISCL	82	1.01e-25	DSPDNSDISN	VVLKYISDMLMEEELESKNFMFEDCLA	LQAAEKSFYD

Table S2. *Cont.*

Motif 12

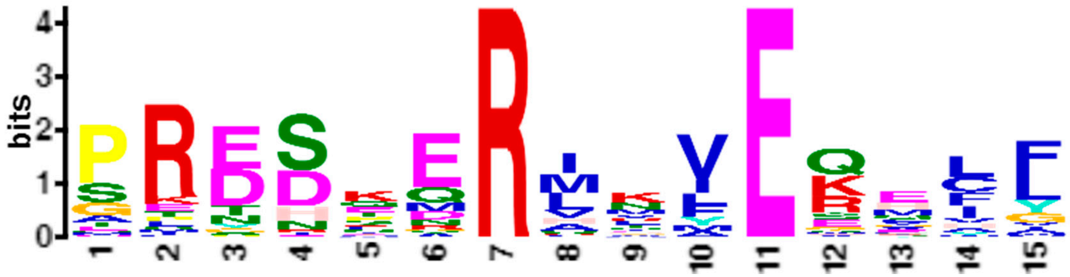

25.	27613.m000642_PAT1	441	9.53e-15	AIFESIDVTL	PRGHKERINVEQHCL	AREVVNIVAC
28.	29949.m000124_PAT1	450	1.15e-14	AMFESIDVTL	SREHKERINVEQHCL	ARDVVNIIAC
27.	29648.m001919_PAT1	461	3.94e-14	AVFESLDATL	PRESQDRMNVEKQCL	ARDIVNIVAC
24.	27529.m000047_PAT1	445	4.96e-12	AMFESIDAGS	SRDDKQRINAEQHCV	ARDIVNMIAC
13.	28650.m000186_LISCL	561	6.38e-12	ALFDMIDSTI	PREEPERMMFEKEVF	GRYAVNVIAC
26.	28166.m001040_PAT1	362	7.22e-12	AMFESIDVRL	PRERKDRIGVEQHCL	AKDIVNIIAC
17.	28650.m000191_LISCL	664	9.24e-12	ALFDMFDTNM	SREDQMRLKFEKEFY	GREALNVIAC
22.	30169.m006306_Os4	442	1.18e-11	IFDSLEASLL	PRNSRQRMKIEKLHF	AEEIRNIVAY
18.	29767.m000200_LISCL	640	1.18e-11	SLFDMLDSIV	PREDMERMLIEKEII	GREALNVVAC
37.	30024.m001746_SCL3	350	1.68e-11	ALFDCLESTV	SRTSLERLKVEKMLF	GEEIKNIISC
12.	27568.m000253_LISCL	640	1.89e-11	TLFDMLETNV	PREIPERMLIEREIF	GWEAKNVIAC
14.	28650.m000187_LISCL	586	3.76e-11	ALFDMFEINV	PREDDQRMLYEKAIF	GRDIMNVIAC
23.	30131.m007029_Os43	587	1.37e-10	AIFDSLDATF	PPDSTQRAKVEQYIF	APEIRNIVAC
39.	29634.m002156_SCL4/7	507	5.49e-10	AVFESLEPNL	SRDSTERLQVERLLL	GRRIAGAVGP
21.	30147.m013794_Os4	428	5.49e-10	AIFDSLDAML	PRYDTRRAKMEQFYF	AEEIKNIVSC
15.	28650.m000189_LISCL	561	6.63e-10	SLFDMFEAIA	TREDQERLVFERELI	GKDVMNVVAC
16.	28650.m000190_LISCL	715	7.99e-10	SLFDMFDSTL	SREDQGRMMLENEIY	GREAMNVVAC
38.	30131.m007244_SCL3	371	9.61e-10	ALFDCLESTV	SRPSIERQKVEKMLL	GEEIKNIIAC
29.	30169.m006225_PAT1	516	1.15e-9	ALFESIESTV	QRDHTERVKVEEGLG	RKLANSVACE
20.	29929.m004746_Os4	408	1.51e-9	AIFDSLDTML	PKYDTKRVKIEQFFY	GEEIKNIVSC
42.	30073.m002204_SCR	715	1.80e-9	ALFDSLGASY	GEESEERHVVEQQLL	SREIRNVLAV
34.	29910.m000940_Rc_GRAS	483	2.15e-9	AYFDCLDACM	ERNDKNRMIMESMYF	GIGIKNMIAT
41.	29872.m000537_SCR	341	3.92e-9	ALFDALGDGL	GIDSVERHTVEQQLF	GCEIRNIVAV
43.	29661.m000901_SHR	342	1.23e-8	IPYDTVDTFL	PRGSKQRQWYEADIC	WKIENVIAHE

Table S2. *Cont.*

32.	29889.m003284_Rc_GRAS	461	1.55e-8	AYFDCFDACM	ERDDPNRMAAESVFF	HHGIRNIVAS
44.	29661.m000923_SHR	414	2.85e-8	VYFESLEESF	SRTSNERLMLERSAG	RAIVDLVACQ
19.	29728.m000844_Os19	354	3.07e-8	AMYDSLDDCL	PLESSERLSIEKNHL	GKEIKSMLNY
5.	30190.m011131_DLT	548	3.07e-8	AIFDSINTSL	PLDSLVRIKIEEMFA	REIRNIVACE
46.	30156.m001710_SHR	342	3.56e-8	IPFDALETFL	PTDSCQRIEYESDIG	HKIENIISYE
40.	28492.m000479_SCR	414	3.83e-8	TLFDSLGAFL	PCDDSSRHRVEHCLL	YREINNVLAI
45.	29706.m001281_SHR	450	4.43e-8	FLDSTSSAFK	GRESEERRVMEGEAA	KALINCGEMN
9.	29908.m006159_HAM	387	2.48e-7	ALYDSLEAGF	PMQSRARALVERVFL	GPRIAGSLDR
33.	29889.m003285_Rc_GRAS	475	4.43e-7	SAYFDCLESC	MKDNSNRMILESLHF	GEGIKNIVAT

Motif 13

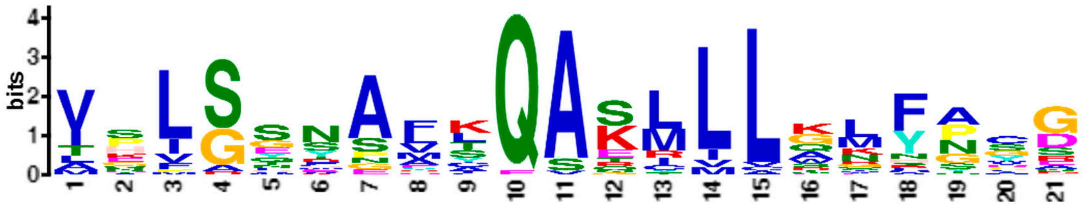

2.	29692.m000540_DELLA	551	1.22e-19	TRLGLAGFAP	VHLGSNAFKQASMLLALFAGG	DGYRVDENNG
1.	28677.m000055_DELLA	514	1.22e-19	TRFESAGFDR	VHLGSNAFKQASMLLALFAGG	DGYRVEENNG
39.	29634.m002156_SCL4/7	559	4.62e-16	ILMESCGFES	VALSHYAMSQAKILLWNYNYS	SLYSLVESQP
23.	30131.m007029_Os43	638	1.61e-15	KLMEGKGFKG	VPLSANAVTQSKILLGLYSCD	GYRLTEDKGC
42.	30073.m002204_SCR	765	5.29e-15	EKLRQSGFKG	ISLAGNAATQATLLLGMFPSD	GYTLVEDNGT
41.	29872.m000537_SCR	391	6.52e-15	NELRRAGFQP	VSLGGNPAAQASLLLGMFPWK	GYTLVEEENG
3.	29807.m000482_DELLA	467	1.09e-14	SRLTAAGFMP	LHLGSNAFKQASMLLTLFSSE	GYCVEENDGC
37.	30024.m001746_SCL3	401	4.79e-14	QRLDLAGFGN	VPLSYCGLLQARRLLQGYGCD	GYRIKDENGC
38.	30131.m007244_SCL3	422	5.27e-14	LRLELAGFGR	VPLSYHGMLQASRLLQSYGYD	GYKIKEENGC
32.	29889.m003284_Rc_GRAS	512	5.27e-14	SFFARFGMIQ	TELSTSSLYQASLVLKKFPCG	SSCTLDVNEK
9.	29908.m006159_HAM	436	3.29e-13	EWSSGAGFRP	VNISFANHCQAKLLLGLFNDG	YRVEELGCNR
33.	29889.m003285_Rc_GRAS	526	4.68e-13	AFFTRFGMLE	TELSTSSLCQAKLIAKKFACG	NACTLSMDGK
34.	29910.m000940_Rc_GRAS	534	1.01e-12	AFFARFGMVE	TDLSSSALLQANLIVKKFACG	NCFTLDRNGK
35.	30076.m004651_Rc_GRAS	579	1.67e-12	AFFSRFRMVE	IGFSESALYQASLVCKQFACG	SSCSLDKNGK
30.	29889.m003281_Rc_GRAS	389	2.51e-12	DYFIRFGMVE	TQVSMSSFYQAELMLKNFASG	KSCLLVRNGK
40.	28492.m000479_SCR	466	2.72e-12	LAARSTSFMQ	VPMSGNSMAQAQLILNMFPPA	HGYSLAQGEG
5.	30190.m011131_DLT	598	1.04e-11	KLMEQGGFRC	MGISEREVLQSQMLLKMYSCE	DYRVKERQDR
19.	29728.m000844_Os19	407	1.34e-10	GRMENHGFSG	MKLSSKSLIQAKLLLKIRTHH	SPPQFNGENS
43.	29661.m000901_SHR	392	3.43e-8	QRMRNANFRS	ISFGEEAVSEVKTMLGEHAAG	WGLKKEEDDL

Table S2. *Cont.*

Motif 14

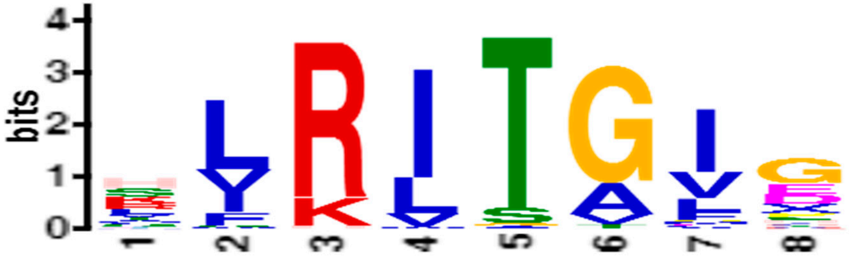

5.	30190.m011131_DLT	420	1.12e-10	SLASRTNPPS	HVRITGIG	ESKQELNETG
38.	30131.m007244_SCL3	185	3.65e-10	TLSARPEGPP	HLRITGIH	EQKEVLEQMA
37.	30024.m001746_SCL3	165	3.65e-10	ALSARREGPP	HLRITGIH	QQKEVLDQMA
12.	27568.m000253_LISCL	504	9.99e-10	RLSSRPGGPP	KLRITGID	FPHPGFRPAE
17.	28650.m000191_LISCL	528	1.47e-9	RLSKREGGPP	KLRITGIE	LPQSGFRPGE
16.	28650.m000190_LISCL	578	1.47e-9	LLSMRDGGPP	KLRITGIE	LPQQGFRPAE
14.	28650.m000187_LISCL	450	1.47e-9	RLSQRPGGPP	KLRITGIE	LPQPGFRPAE
15.	28650.m000189_LISCL	425	2.60e-9	HLSTRPGGPP	KVRITGID	YPQPGFRPGE
27.	29648.m001919_PAT1	325	6.99e-9	TLANQPGKPP	HLRLTGID	DPESVQRSTG
28.	29949.m000124_PAT1	314	7.58e-9	AFAARPGGPP	RIRITGID	DSTSAYARGG
34.	29910.m000940_Rc_GRAS	355	1.00e-8	LVSRCDCPLE	HLKITAIG	TTSRELIENT
25.	27613.m000642_PAT1	305	1.53e-8	ALAARPGGPP	HVRLTGID	DSTSAYARGG
20.	29929.m004746_Os4	281	3.09e-8	LANRPDKKPR	CLRITGVG	NSAERLQALG
3.	29807.m000482_DELLA	284	4.33e-8	ALALRPGGPP	LLRLTGIG	PPSPDGRDSL
23.	30131.m007029_Os43	463	4.92e-8	ALAARPGGAP	FLRITGVG	SCIESVRETG
21.	30147.m013794_Os4	301	6.74e-8	LANRAGKPPC	RLRITAVG	LCVGRFQTIG
19.	29728.m000844_Os19	229	6.74e-8	SEKASSSNRI	SLRITGLG	RSLEELQETE
24.	27529.m000047_PAT1	309	1.83e-7	ALADRPGGPP	AIRITGVD	DPQSAYARGG
2.	29692.m000540_DELLA	370	2.04e-7	ALALRPGGPP	AFRLTGIG	PPSHDNSDHL
1.	28677.m000055_DELLA	330	2.04e-7	ALALRPGGPP	AFRLTGIG	PPQSNNTDAL
4.	30170.m013590_DELLA	221	2.44e-7	DLAARPGGPP	TIRISGIG	FPNHDNSDYL
13.	28650.m000186_LISCL	425	2.92e-7	DLSTRHGGPP	RLHITGIE	FPQPGFRPAE
44.	29661.m000923_SHR	264	5.36e-7	ALATRTDETP	HLRLTTIV	ASKTNGGGGG
33.	29889.m003285_Rc_GRAS	348	6.32e-7	LVSDFDCNLE	LLKITAVG	TTSKHLIKDT

Table S2. *Cont.*

6.	29646.m001070_HAM	314	6.90e-7	DHFCNKLTSP	VLRITAVV	TEDYAIQTQL
9.	29908.m006159_HAM	251	7.43e-7	VSRKDGPPTP	HLRITALS	RGGNSGKRSI
41.	29872.m000537_SCR	217	8.06e-7	ILASRSKKIR	SMRITGFG	SSSELLESTG
40.	28492.m000479_SCR	291	8.06e-7	ILATRMEGPP	HIRMTGMG	TSMDLLVETG
22.	30169.m006306_Os4	309	8.74e-7	TLASRPEGPP	KVRITGLI	NDHQNLLELE
39.	29634.m002156_SCL4/7	374	1.03e-6	LATRSAGRPL	SIRISGIP	APVLGKSPAA
7.	29820.m000985_HAM	280	1.10e-6	GYNQRHLQQI	TLKITAIK	WREEHPWRKL
31.	29889.m003282_Rc_GRAS	252	1.50e-6	ENVAEANRSH	LLKITAVE	TTSKHLIEET
42.	30073.m002204_SCR	592	1.74e-6	ILASRPGGPP	YVRLTGLG	TSIEALEATG
45.	29706.m001281_SHR	306	1.86e-6	LTRRSGGPPP	LVRITVIT	ATVESDQNTE
35.	30076.m004651_Rc_GRAS	400	2.01e-6	LAERQQRPLE	HLKVTALG	LTGKGKKIEE
30.	29889.m003281_Rc_GRAS	210	2.49e-6	LANRQECPIE	LLKISGVG	VKASRQRIED
29.	30169.m006225_PAT1	382	3.78e-6	LSERLNGKPA	TVKITAVA	DNSAEEKERL
11.	30174.m008828_HAM	510	5.30e-6	ELALRNGGVS	SLKITAFV	SPSHDEIELG
46.	30156.m001710_SHR	184	3.18e-5	ALAKRPEGPP	SLRITVPF	TRPQVPPWLN
8.	29820.m000986_HAM	481	4.24e-5	ETVKEKQSLY	KVRIGGEW	NNEMILEWRG
32.	29889.m003284_Rc_GRAS	150	1.38e-4	GSSRGLSTEE	VIRIAGAR	FIQSCWQAVD
				Motif 15		
				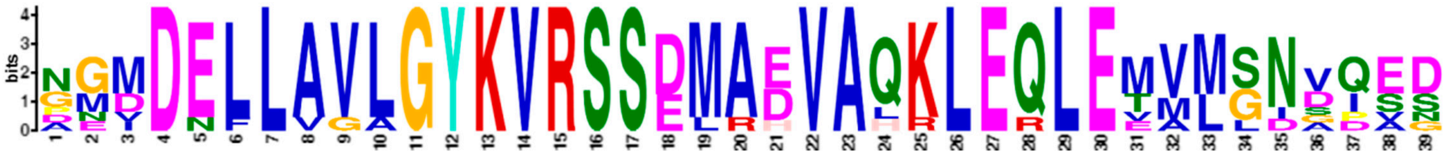		
2.	29692.m000540_DELLA	54	5.80e-46	IWEDEEAKGD	GGMDELLAVLGYKVRSSDMAEVAQKLEQLEEVMGNVQED	GLCQLASETV
1.	28677.m000055_DELLA	37	5.73e-44	EEEEQQQQDG	GGMDELLAVLGYKVRSSDMAEVAQKLEQLEMVMGIAQED	GISHLSDTVH
3.	29807.m000482_DELLA	27	1.37e-27	SSSSSITNKP	PEIDNLLAGAGYKVRSSDLRHVAQRLERLETAMLNSPSS	HLSVLASDTV

Table S2. *Cont.*

Motif 16

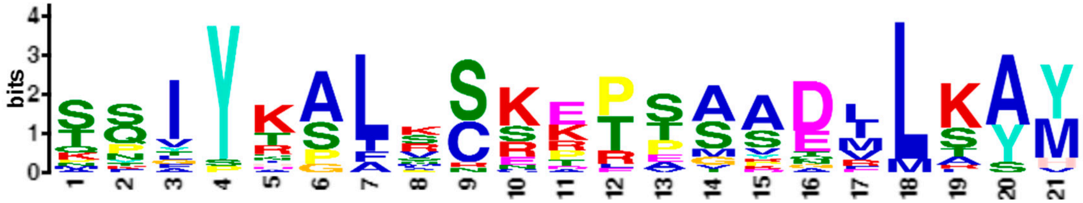

28.	29949.m000124_PAT1	234	1.06e-19	GLVARLASSG	SSIYKSLRCKEPASADLLSYM	HILYEVCPYF
18.	29767.m000200_LISCL	425	1.34e-18	GLDARLAGTG	SQIYKGLVSKRTSAANVLKAY	HLYLAACPFR
25.	27613.m000642_PAT1	225	1.96e-18	GLVARLASSG	SSIYRALRCKEPASAELLSYM	HILYEVCPYF
12.	27568.m000253_LISCL	424	4.09e-18	GLEARMAGSG	TQIYKAFMSRPTTAADVLKAH	HLFLAACPFR
17.	28650.m000191_LISCL	448	4.59e-17	GLEARLAGTG	AQIYTALSSEKLSAADMLKAY	LAYISACPFN
27.	29648.m001919_PAT1	245	3.41e-16	GLAARMAASG	KYLYKALKCKEPPSSDRLAAM	QILFEICPCF
26.	28166.m001040_PAT1	256	5.11e-16	GLVARKESSG	NNIYHALRCREPESKDLLSYM	QILYEICPYL
24.	27529.m000047_PAT1	229	3.21e-15	GLRARVELSG	SKIYRALKCEAPVSSDLMTYM	GILFKICPYW
16.	28650.m000190_LISCL	498	3.62e-14	ARLAGSVTGM	QSFYTSLASRRRTAADILRAY	KTHLHACPFK
14.	28650.m000187_LISCL	370	3.37e-13	ALETRLAGTG	TPAYSPLLSSKTPVSDILKAY	QVYVKACPFK
13.	28650.m000186_LISCL	345	2.49e-12	GLEARLAGTG	TPGYAPAVNSTTSAAGMLKAY	HAYTTACPFQ

Motif 17

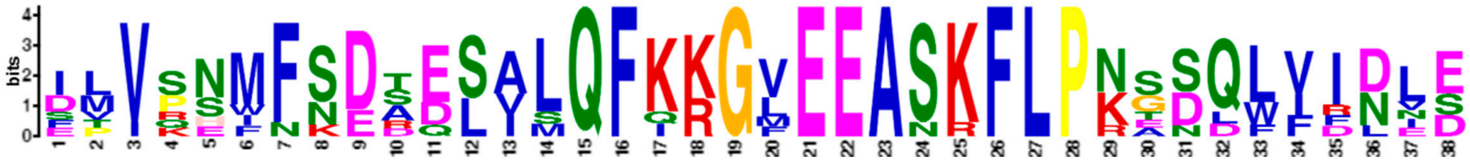

16.	28650.m000190_LISCL	278	2.77e-38	GDGIMGSSPS	EMVQNMFSDTDSVLQFKRGLEEASKFLPRASQLVIDLE	SNSFANGQKE
17.	28650.m000191_LISCL	230	1.95e-31	HDVMGSFESK	IVVPNFFSERELALQFQRGVEEANRFLPKENQLVIDLE	TNASIPEMKE
12.	27568.m000253_LISCL	211	4.65e-28	AADGLVDSPL	STISEIFSDSESILQFKKGFEEASKFLPNGSLFIDLES	TGLFLKDLRE
18.	29767.m000200_LISCL	206	9.34e-27	DGLVHSPNST	FPVPHWNNESQSISQFIKGVEEASKFLPNGDDLFRNIE	VNRFLFKEPK

Table S2. *Cont.*

Motif 18

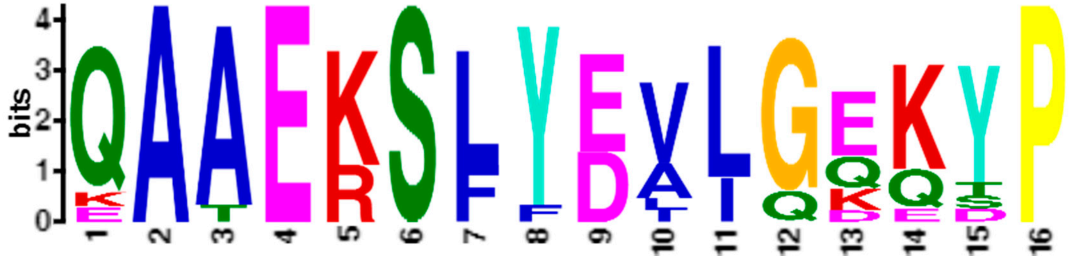

15.	28650.m000189_LISCL	97	3.50e-20	**PCMLQDCLAL**	**QAAEKSLYDVLGEKYP**	**PICDQSPSSI**
13.	28650.m000186_LISCL	110	8.30e-20	**NFMFEDCLAL**	**QAAEKSFYDVLGQKYP**	**ASLDQSPFLD**
18.	29767.m000200_LISCL	110	1.06e-19	**TCMLQDSLDL**	**QAAEKSFYEVLGKKYP**	**PSPEPKRVSI**
17.	28650.m000191_LISCL	112	2.48e-19	**PCMFHDPLAL**	**QAAERSLYDVLGEKYP**	**SSPNQSSSFG**
14.	28650.m000187_LISCL	105	1.44e-18	**TCMLQDCLAL**	**QAAEKSLYDVLGQEYP**	**HSLSHCPQIV**
12.	27568.m000253_LISCL	109	2.73e-18	**CMFQESSAAL**	**QAAEKSLYELIGEKYP**	**PSINYDSAAH**
16.	28650.m000190_LISCL	139	2.98e-15	**PPMYYDPLDL**	**KATEKSLYDVLGEQDP**	**SSLESPQPYI**

Motif 19

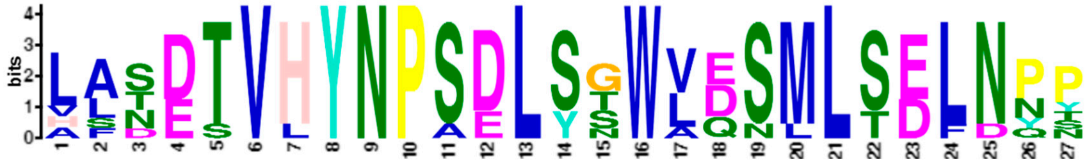

	**Name**	**Start**	***P*-Value**		**Sites**	
2.	29692.m000540_DELLA	97	6.38e-30	**GNVQEDGLCQ**	**LASETVHYNPSDLSTWLESMLSELNPN**	**PNFDPSQQPD**
1.	28677.m000055_DELLA	79	1.18e-29	**MGIAQEDGIS**	**HLSDTVHYNPSDLSGWVQSMLSELNNP**	**LDTIQNQQDS**
3.	29807.m000482_DELLA	70	1.20e-23	**LNSPSSHLSV**	**LASDTVLYNPSDLSSWVDSLLTEFNQS**	**QPLPSLPPDL**

Table S2. *Cont.*

Motif 20						
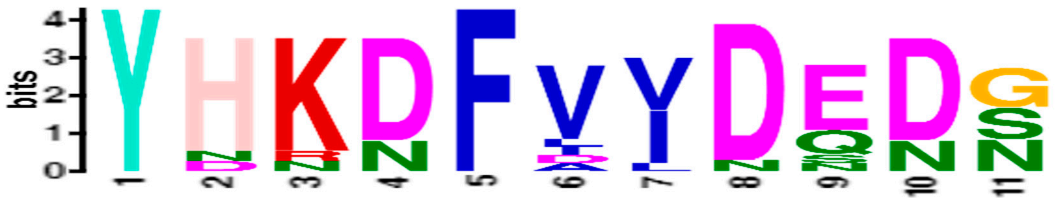						
16.	28650.m000190_LISCL	784	3.86e-15	EKCRHKLKTW	YHKDFVIDEDN	NWMLQGWKGR
12.	27568.m000253_LISCL	709	1.43e-14	AAAKEKVNAL	YHKDFVIDEDS	RWLLQGWKGR
14.	28650.m000187_LISCL	655	6.70e-14	KKVRCTVRSE	YHKDFVVDENG	RWMLQGWKGR
15.	28650.m000189_LISCL	629	1.72e-13	VKRVRNIKND	YHKDFAVDEDG	HWMLMGWKGR
17.	28650.m000191_LISCL	733	1.54e-12	KKLKCRVKEG	YHNDFVVDQDG	QWMLQGWKGR
13.	28650.m000186_LISCL	630	4.07e-11	KKVITTVKSN	YNKNFIVDEDS	QWMLQGWKGR
18.	29767.m000200_LISCL	709	4.78e-11	KQAIEKVRKL	YHKNFLINEDG	RWLLQGWKGR

Table S3. Information of all primers used in this study.

Gene Name	Forward (from 5′ to 3′)	Reverse (from 5′ to 3′)
28677.m000055	TCAAAAGCTTGAACAGCTTGAA	ACCAGGAATGGCTCTAAGATCA
29692.m000540	GGTTGGAAATTGGCTCAGTTAG	CGCTTCTTGCTCAACGATAGTA
29807.m000482	CATTTGTTGATGACATGTGCTG	GGACACGCCTCATAAAAGTGAT
30170.m013590	TTTCGGATTTAGCCAAGTCAAT	AAATATTGGGGGTAGAGCCTGT
30190.m011131	CAGGCTCCTTTCTCTCTGACAT	TCCACAACATTCTCACTTGGTC
29646.m001070	GAGGATTCCACTCCTACGCTAA	CTGCTCGGATAAGCTCTTGAAT
29820.m000985	TGGCTCTTTCTCAACAACAAAA	AGGCTAGCCATTCTTTTCTTCC
29820.m000986	CGGTCTCAAATTAAAGGTGGAG	CATCCCCATCTCCAAAAGTAAG
29908.m006159	ACTTGCAACTCAAACCCAAACT	TTGCAAGGCGTCAGTAAAATAA
29916.m000544	AAGAAAGGATGAATGGGCTACA	ATCTACAATCCCTAACCCAGCA
30174.m008828	CCTTGAATTCTGGTTCTTGGTC	GCTTCCAGTGATTCAAGTAGGC
27568.m000253	TAGCACAGAAGTGGGACACAGT	AGTAGTGGAAAACAGCCTCTCG
28650.m000186	ATTAGCACATTTCTTCGCCAAT	AGGCCATTGAAAACCATAAAGA
28650.m000187	ACAATGCACCATTCTTTGTCAC	TGATCTAGTGAGAGCTGCCTGA
28650.m000189	ATCTGCAGCCTTTTAGATCGAG	AGAAGAAGGGGATTGATCACAA
28650.m000190	ATCAAATGGTGGAAAGACTCGT	TAAAAGCTTTGCATTCCAGTGA
28650.m000191	AGGTGGTTGTGAAGGAAGAGAA	TGTAGTGCCTTGTTTGATCCAC
29767.m000200	TCGTAACATTGAGGTCAACAGG	TTACCCTCCCCAGCACTATAAA
29728.m000844	TGGCAATCGTTGATACAGATTC	AGCTGTGGCTACATTGTTCTCA
29929.m004746	ATCGAAGCTATGGAAGAAGCAG	AAGTAGCGTAAGCCGATCAGAC
30147.m013794	CGAGCTCTAAGTTTCGCAGAAT	TCTCCATGATTTTCACCTTCCT
30169.m006306	AATCCTCCAAGCAATCAAGAGA	GCCTCTCAATCCTATCACAACC
30131.m007029	AGCTGGATGGAGATGTTGAAGT	TGGACTCTGTCCTTTCCCTTTA
27529.m000047	GAGCAAATGGTGTCAGTCTCTG	AATCCTAGGCTCGTATTCCACA
27613.m000642	AGGATGGATCCAATCAAATCAC	AGAACTTCCTGAAGATGCCAAC
28166.m001040	TCCACCAGACAATCTAAAGCAA	TCTGCATGTACGAAAGCAAATC
29648.m001919	GTGAATTTTGCTTTCCAACTCC	TTTCAACATTCATCCTGTCCTG
29949.m000124	GCATTCGTATTACAGGCATTGA	TGGTTCTGAGTGCTGACACTTT
30169.m006225	TGTTTGCCAAAGAACATATTGC	TATTATCCGCAACAGCTGTGAT
29889.m003281	TTTTGAGCACATGAAGATGGAC	TCTTGGTGCAATTCTTCTTTGA
29889.m003282	CAAGCTGCTGAAATTCAAAATG	ACTGATCACAAAACTCGAGCAA
29889.m003284	TTCACTGCAATCCAAGAAATTG	TGACTGCCCTAAAAGAGAAAGG
29889.m003285	TGACTGCAATCTGGAGCTTTTA	AGAATTTAGCCTATCGGGCAGT
29910.m000940	TTTGCTGAAGCTCTCCATGATA	GTCCATTGCATTCCATATCTGA
30076.m004651	TATGTTCTGAATATGGCGCTTG	GTTGAGCAGCTCCAAAGAAGAT
29790.m000809	TGGGAAGTGAAGATAGGATGGT	CATGCCAACAAATCATCAAACT
30024.m001746	ACTTGTGGAAATCATGTTGCTG	TCAAGAATGGAAACATCTCGAA
30131.m007244	GCCTTGAGCATATCTCTCACCT	ATTGCTTCTACAATGGCCTGAT
29634.m002156	GTGAACATGGAGATCCAACTGA	AACGATCCCAAAATCCACTATG
28492.m000479	TGTTTCACATCCTGGCTACAAG	CCTGTTGCATCGTACAGTGAAT
29872.m000537	GAGACTCCTCGGTCTTTTGCTA	GGAAAGCGTTGAAGATTTTCTG
30073.m002204	AGCTACTTTGCTTCTTGGGATG	TCAACAAAATGGTATTGGCAAG
29661.m000901	CCACAAACTCATCAAATCCGTA	TCATACCATTGTCTTTGCTTGC
29661.m000923	TGTGGATGCTTAATGAACTTGG	TCAAATGCTTCCATTATTGCAC
29706.m001281	ATGAGGTTAGTCCTTGGTTTGC	ACTGAAAATGGGGTCTCAGTGT
30156.m001710	TCTTGGTAGTCTTGATGGAGCA	ACTCATTTGCCTCCTTTGGATA
